# The Rapid Evolution of De Novo Proteins in Structure and Complex

**DOI:** 10.1093/gbe/evae107

**Published:** 2024-05-16

**Authors:** Jianhai Chen, Qingrong Li, Shengqian Xia, Deanna Arsala, Dylan Sosa, Dong Wang, Manyuan Long

**Affiliations:** Department of Ecology and Evolution, The University of Chicago, Chicago, IL 60637, USA; Division of Pharmaceutical Sciences, Skaggs School of Pharmacy and Pharmaceutical Sciences, University of California San Diego, La Jolla, CA 92093, USA; Department of Cellular & Molecular Medicine, School of Medicine, University of California San Diego, La Jolla, CA 92093, USA; Department of Ecology and Evolution, The University of Chicago, Chicago, IL 60637, USA; Department of Ecology and Evolution, The University of Chicago, Chicago, IL 60637, USA; Department of Ecology and Evolution, The University of Chicago, Chicago, IL 60637, USA; Division of Pharmaceutical Sciences, Skaggs School of Pharmacy and Pharmaceutical Sciences, University of California San Diego, La Jolla, CA 92093, USA; Department of Cellular & Molecular Medicine, School of Medicine, University of California San Diego, La Jolla, CA 92093, USA; Department of Ecology and Evolution, The University of Chicago, Chicago, IL 60637, USA

**Keywords:** de novo genes, gene duplicates, structural evolution, protein complex, new genes

## Abstract

Recent studies in the rice genome-wide have established that de novo genes, evolving from noncoding sequences, enhance protein diversity through a stepwise process. However, the pattern and rate of their evolution in protein structure over time remain unclear. Here, we addressed these issues within a surprisingly short evolutionary timescale (<1 million years for 97% of *Oryza* de novo genes) with comparative approaches to gene duplicates. We found that de novo genes evolve faster than gene duplicates in the intrinsically disordered regions (such as random coils), secondary structure elements (such as α helix and β strand), hydrophobicity, and molecular recognition features. In de novo proteins, specifically, we observed an 8% to 14% decay in random coils and intrinsically disordered region lengths and a 2.3% to 6.5% increase in structured elements, hydrophobicity, and molecular recognition features, per million years on average. These patterns of structural evolution align with changes in amino acid composition over time as well. We also revealed higher positive charges but smaller molecular weights for de novo proteins than duplicates. Tertiary structure predictions showed that most de novo proteins, though not typically well folded on their own, readily form low-energy and compact complexes with other proteins facilitated by extensive residue contacts and conformational flexibility, suggesting a faster-binding scenario in de novo proteins to promote interaction. These analyses illuminate a rapid evolution of protein structure in de novo genes in rice genomes, originating from noncoding sequences, highlighting their quick transformation into active, protein complex-forming components within a remarkably short evolutionary timeframe.

**Significance**

The structural evolution of de novo proteins remains a fundamentally important question for understanding the evolution of molecular functions of de novo genes. We detected a rapid evolution of protein structure in de novo genes of *Oryza* on a surprisingly short timescale.

## Introduction

The complexity and adaptability of biological functions often find their roots in the ever-evolving genetic systems. Important to this is the emergence of de novo genes (Long et al. 2003; Alba and Castresana 2005; Levine et al. 2006; McLysaght and Hurst 2016)—genes that arise from regions of DNA once categorized as the “junk” that used to be considered functionally insignificant (Ohno 1972; Fagundes et al. 2022). The birth of de novo genes was deemed impossible or functionally irrelevant (Jacob 1977; Mayr 1982). However, recent studies in rice, flies, yeast, fishes, and mammals, with reports of many candidate de novo genes, have challenged this dogma and provided concrete evidence that de novo genes can indeed emerge from noncoding sequences through a stepwise mutational process, contributing to increased protein diversity (Knowles and McLysaght 2009; Zhao et al. 2014; Xie et al. 2019; Zhang et al. 2019; Zhuang et al. 2019; Heames et al. 2020; Vakirlis et al. 2022; An et al. 2023; Montañés et al. 2023). Despite these progresses, our understanding of these novel proteins, particularly their structural characteristics at the secondary, tertiary, and complex levels, and the rate of their structural evolution, remains largely unexplored.

Gene duplicates have long been recognized as a predominant source of new gene functions (Long et al. 2013). These duplicates retain sequences from their parent genes and contribute to phenotypic evolution through various mechanisms, including neofunctionalization, hypofunctionalization, subfunctionalization, and gene dosage regulation (Ohno 1970; Kaessmann 2010; Birchler and Yang 2022). In contrast, de novo genes evolve through nonduplication mechanisms and have been shown to play diverse roles in biological functions. Their contributions have been highlighted in multiple systems, for example, DNA repair in yeast (Cai et al. 2008), providing a novel antifreeze function in Arctic fish (Zhuang and Cheng 2021), diversification of rice morphology (Chen et al. 2023), flora transition in *Arabidopsis* (Takeda et al. 2023), cortical expansion in humans (An et al. 2023; Qi et al. 2023), and even oncogenesis in human cancers (Suenaga et al. 2014). The emergence and functional diversity of de novo genes introduce a novel dimension to our understanding of genome evolution and functional innovation, expanding our knowledge beyond traditional gene duplication models (Knowles and McLysaght 2009; Carvunis et al. 2012; Zhao et al. 2014; Zhang et al. 2019; Vakirlis et al. 2022; Broeils et al. 2023).

Due to their relatively recent origins, it can be hypothesized that de novo proteins may not have evolved into well-folded structure (Bornberg-Bauer et al. 2021). This would lead to a characteristic feature: a lack of stable tertiary structure when isolated, thus manifesting as intrinsic structural disorder (ISD) in intrinsically disordered regions (IDRs) or regions of random coils. It is found that vertebrate species with a higher codon adaptation index score evolve more ISD domains (Weibel et al. 2023). ISD is also commonly found in proteins related to human genetic diseases (Midic et al. 2009; Vavouri et al. 2009). Despite advancements in functional studies of ISD proteins, the extent of ISD in de novo genes remains a subject of debate. Several studies suggest a strong tendency toward ISD in de novo genes or newly evolved domains (Bitard-Feildel et al. 2015; Basile et al. 2017; Wilson et al. 2017; Heames et al. 2020; Lange et al. 2021; Heames et al. 2023). Conversely, other studies present inconsistent results due to different average disorders in different species (Ekman and Elofsson 2010; Schmitz et al. 2018; Vakirlis et al. 2018). The question of whether ISD is influenced by gene age or if it can evolve over time remains unresolved.

Additionally, the evolvability of well-folded structural elements in de novo genes, such as, 3_10_ helices, α helices, and β strands, remains an open question. Are the amino acid compositions of de novo proteins optimized for structural stability over time? Recently, AlphaFold2 stands as the leading deep learning tool for predicting protein structures utilizing coevolutionary information from multiple sequence alignments (Jumper et al. 2021). MD (molecular dynamics) simulation studies have revealed that most de novo proteins are flexible in structure and a minority of them adopt well-known protein structures (Middendorf and Eicholt 2024; Peng and Zhao 2024). Despite the tendency of de novo proteins to be disordered with few (or no) orthologs, AlphaFold2's predictions reveal that they generally achieve higher-confidence scores per residue (predicted local distance difference test [pLDDT]) than random sequences (Middendorf et al. 2024). The AlphaFold2 performs the MD refinement (called “relax” in AlphaFold2 terminology) using OpenMM (Jumper et al. 2021). In addition, a benchmarking study based on 2,613 proteins with experimentally determined structures indicates that AlphaFold2 is a good predictor of the structure of loop regions (regions of neither α helices nor β strands), especially for short loop regions (Stevens and He 2022). The pLDDT score is an excellent metric for assessing modeling confidence, disorder levels, and structural variability (Saldaño et al. 2022; Wilson et al. 2022), with AlphaFold2 demonstrating a significant correlation between pLDDT scores and the presence of secondary structures in disorder-rich proteins, both globally and locally (Wilson et al. 2022). Recent studies showed that model quality can be estimated by generating many structure models for the same protein and quantifying the structural similarities among the models by TM (template modeling) score (Mukherjee and Zhang 2009; Peng and Zhao 2024). These findings suggest AlphaFold2's pivotal role in elucidating the biological implications of de novo proteins, which are predominantly characterized by variable structural changes.

Another rising question is whether or how de novo proteins, which are often very short, interact with other usually larger proteins and their ability to form complexes with other biomolecules. Indeed, roughly 40% of all protein–protein interactions are between proteins and shorter peptides, many of which play critical roles in cellular life-cycle functions (Lee et al. 2019). Recent advances like AlphaFold-multimer excel in predicting peptide–protein interactions (Johansson-Åkhe and Wallner 2022), which could facilitate our understanding on the evolution of de novo protein and potential conformational changes upon binding. Evaluation of AlphaFold-multimer predictions has revealed that highly confident structures could be obtained from AlphaFold-multimer even for proteins without homology to any existing structures (Zhu et al. 2023).

The structural evolution of proteins is conventionally perceived as a slow process, maintaining remarkable conservation over hundreds of millions to billions of years, contrast to the rapid changes observed in their primary structure (Ingles-Prieto et al. 2013; Liljas et al. 2016). In this study, we explore the evolutionary patterns of de novo genes with a focus on their protein structures and complexes, taking advantage of a large number of de novo genes identified in *Oryza* genomes with clearly reconstructed origination processes from noncoding ancestral sequences in intergenic regions (Zhang et al. 2019). We analyzed multiple properties of protein structure including proportions of IDRs, secondary structure elements (including the unstructured random coils and structured α helices and β strands), amino acid composition and properties (such as charges, weights, and hydrophobicity), molecular recognition features (MoRFs), and the protein complexes. We revealed the rapid evolution of these *Oryza* de novo proteins in forming structures and complexes due to their different features from duplicated proteins, showing their rapid assembly into new protein complex with previously existing old genes. These insights challenge the conventional view of slow structural evolution of proteins and have revealed a dynamic world of protein evolution over a surprisingly short evolutionary period (<1 million years).

## Materials and Methods

### Gene Age Dating and Data Sources

The de novo gene list and origination branches (ages) were retrieved from a previous study (Zhang et al. 2019), which was based on the synteny alignment between focal species *Oryza sativa japonica* (br1) and outgroup species. Based on the *Oryza* phylogenetic tree, the 11 species were assigned to six age groups for de novo genes: *Oryza rufipogon* (br2), *O. sativa* subspecies *indica* and *Oryza nivara* (br3), *Oryza glaberrima* and *Oryza barthii* (br4), *Oryza glumaepatula* (br5), and *Oryza meridionalis* (br6). The divergence time was based on the previous report (Stein et al. 2018). The gene duplicates were identified based on BLASTP comparison of genome-wide protein sequences (-evalue 0.001 -seg yes). The gene ages for these genes were determined with a two-step synteny-based method: (i) the reciprocal best orthologous genes were exhaustively searched between focal species and outgroup species, and (ii) the gene synteny blocks were then constructed based on a criterion of no more than five genes within the range of reciprocal best pairs. Due to the higher number of duplicated genes, the groups were further extended into another three branch groups, which are *Oryza punctata* (br7), *Oryza brachyantha* (br8), and *Leersia perrieri* (br9).

### Gene Coexpression Analysis

The genome reference and gene annotations (v66) were downloaded from the Gramene database (http://ftp.gramene.org/oge/release-current/; Gupta et al. 2016). All RNA-seq short-read data sequenced with the Illumina platform for *O. sativa japonica* were downloaded from the National Center for Biotechnology Information Sequence Read Archive database (∼400-GB bases, 2023 August 25; supplementary table S6, Supplementary Material online). We filtered the samples with fastp (Chen et al. 2018) and mapped cleaned reads to the genome reference using STAR v2.7.0a (Dobin et al. 2013). The expression level for all genes and isoforms was measured with RNA-Seq by Expectation-Maximization (Li and Dewey 2011). Since coexpression analysis often involves the relationships between genes across multiple samples, transcript per million was chosen to measure expression because it is commonly used for intersample comparisons. The gene coexpression was analyzed with the Pearson test. We defined the coexpression gene partners as the top 30 coexpressed genes with significant interaction signals for each de novo gene (*P* < 10^−5^). We also randomly picked up duplicated genes for comparison (180).

### The ISD Prediction Based on Sequences

The ISD of protein-coding genes for rice genome (http://ftp.gramene.org/oge/release-current/; Gupta et al. 2016) was analyzed with metapredict (v2.3), a deep learning-based consensus predictor (Emenecker et al. 2021). ISD proteins were defined as proteins with 100% of residues in disordered states (Threshold 1). The ISD level or proportion was evaluated with the fraction of ISD segment out of the full length of a protein. We performed a linear regression analysis on the median ISD levels of proteins across different evolutionary stages, using the “lm” function in the R platform (Racine 2012; R Core Team 2013), to assess their relationship with evolutionary time. We also used AUCpreD (Wang et al. 2016) to identify ISD of de novo genes with the default parameters.

### The Analyses for Evolutionary Changes of the Secondary Structure

We first generated the 3D structures of de novo proteins using AlphaFold2 with default parameters and then extracted the structural elements using STRIDE (Heinig and Frishman 2004; Jumper et al. 2021). For gene duplicates, we randomly picked 30 genes from each branch. We also analyzed the pattern of duplicated proteins using AlphaFold2 public data for rice (UP000059680_39947_ORYSJ_v4.tar). Considering genome version differences between our analyzed data set (International Rice Genome Sequencing Project identifier) and the AlphaFold2 (the identifier of the Michigan State University Rice Genome Annotation Project), we converted the identifiers of the two data sets with strict parameters of BLASTP, including the reciprocal best hits, identical protein sequences (100%), identical lengths, and reciprocally only one match. To elucidate the evolutionary dynamics of protein structure, we quantified the proportion of unstructured (random coil) and structured (α helices and β strands) regions in both de novo genes and gene duplicates (*P*_2nd-structure_). These proportions are defined by the equations:


$$P_{i}=l_{i}/l_{\mathrm{total}},$$

where *i* represents coil, α helix, 3_10_ helix, or β strand, the *l_i_* is the cumulative length of each element *i*, and *l*_total_ denotes the total protein length. The median values for *P_i_* were used to conduct linear regression against the evolutionary time with R platform. For the model without significant linear model support, we also explored nonlinear model based on logarithmic unit of time (log_10_*t*).

MoRFs are prevalent components found within disordered regions of proteins, which could transform from a disordered to an ordered state when they bind to their respective protein partners. We predicted the MoRFs using fMoRFpred and compared their proportions between gene duplicates and de novo genes (Yan et al. 2016). The online tool of ipc2 was used to evaluate isoelectric point and molecular weights (Da) for all de novo genes and 200 duplicated genes randomly selected (Kozlowski 2021). The hydrophobicity scores were estimated with the previously reported method (Wilson et al. 2017).

### The Analyses of Protein Complex Based on AlphaFold2-Multimer

We further classified protein 3D structures based on AlphaFold2 into three groups. The high-confidence potential folding was defined as at least one element over ten amino acids with pLDDT ≥ 0.9 (expressed as the fraction of the maximum 100). The medium-confidence folding was defined as at least one element over ten amino acids with pLDDT ≥ 0.7. Others are defined as low-confidence folding. To understand whether the folding conformation could be changed upon protein binding, we chose both high-confidence folding and low-confidence folding genes and their potential protein partners to conduct protein–protein docking analysis with AlphaFold2-multimer (Evans et al. 2022). The protein partners were chosen based on the following criteria: (i) low percentage of disordered regions (<5%), (ii) highly correlated expression pattern (coexpression correlation coefficient > 0.8), (iii) partner sequence between 200 and 500 amino acids, and (iv) partner as a relatively old gene (br6 to br9). The similarities among resulting models from AlphaFold2 and AlphaFold2-multimer were estimated with USalign (Zhang et al. 2022). The criteria for distinguishing similar folds from random folds are set at TM scores of 0.5 and 0.17, respectively, based on previous reports (Mukherjee and Zhang 2009; Zhang et al. 2022).

### The Analyses of Binding Free Energy and the Dissociation Constant for Complexes

The binding free energy and the dissociation constant were estimated with PRODIGY (Vangone and Bonvin 2015; Xue et al. 2016). The spontaneity and stability of the binding process for protein–protein interactions were evaluated with the change in Gibbs free energy (Δ*G*) and the dissociation constant (*Kd*). The cutoff Δ*G* = −10 kcal/mol (*Kd* of 10^−8^ M) was used to indicate high affinity (Yugandhar and Gromiha 2014; Nikam et al. 2023). Generally, a lower *Kd* value (<1) and a very negative Δ*G* indicate a more stable and tightly bound complex (supplementary fig. S6b, Supplementary Material online). Because the residue–residue (RR) pairs or contacts could occur between a residue in one protein and multiple residues of its partner, we counted RR as both raw numbers and nonredundant ratios. The raw numbers were based on number of total RR pairs estimated with the tool PRODIGY, while the nonredundant ratios were estimated by focusing on unique pairs and adjusted with total protein length of complex.

## Results

### The Levels of ISD in De Novo Proteins Reduce Gradually Over Evolutionary Time

**Fig. 1.**
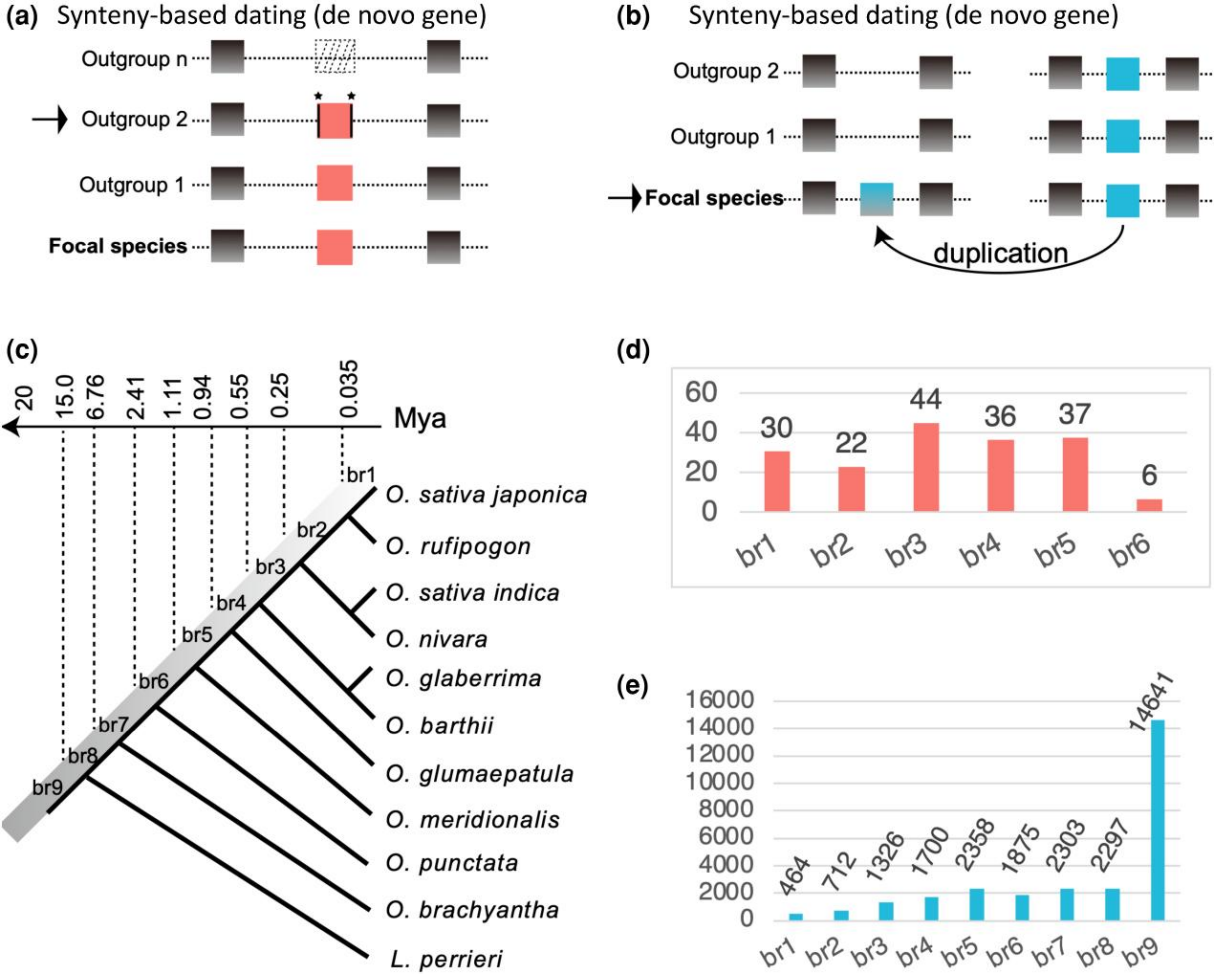
The methodology of gene age dating and number of genes with gene age information for de novo genes and gene duplicates. a) The conceptual diagram for dating de novo gene ages, based on our previous synteny-based method including steps of noncoding outgroups, homology detection failure, and targeted proteomics (Zhang et al. 2019). The dotted box indicates noncoding sequence with DNA level similarity to de novo genes. The neighboring genes are represented in green and blue, with Outgroup 2 as the origination branch of open reading frame. The emergence of the gene is attributed to “trigger” or “enabler” mutations, including substitutions and/or insertions/deletions (indicated by asterisks), as detailed in Zhang et al. (2019). b) The age dating of duplicated genes involves the synteny-based method by identifying the reciprocal best hits for proteins and conserved neighboring reciprocal best hits. The direction of duplication is indicated by an arrow. The emergence of the purple gene is determined based on the presence or absence of conserved synteny in the focal species. c) The phylogenetic framework (br1 to br9) and the corresponding divergence time (million years ago, Mya), which are based on the previous report (Stein et al. 2018). d, e) The numbers of de novo genes and gene duplicates with different ages across the evolutionary branches.

We retrieved all de novo genes previously identified in *Oryza* genomes, which showed a detailed stepwise process of de novo gene origination from ancestral noncoding intergenic regions (Zhang et al. 2019;  Fig. 1a). The gene ages are defined as the branches of open reading frame origination, following the removal of potential gene duplicates with stringent criteria (*e*-value 0.01) against complete nonredundant complete proteome (nr database; Zhang et al. 2019). Synteny-based method could provide strong evidence for de novo origination (Weisman et al. 2020). We locally inferred gene ages based on the synteny-based method for 27,673 duplicated genes (Long et al. 2013), which account for 71.41% of genomic protein-coding genes (IRGSP-1.0.75 version of rice genome; Fig. 1b). Both gene duplicates and de novo genes were assigned into evolutionary age groups from young to old evolutionary epochs based on reported phylogenetic age groups (Zhang et al. 2019;  Fig. 1c and supplementary table S1, Supplementary Material online). In detail, the nine evolutionary age groups cover ∼15 million years of *Oryzeae* evolution, which includes species of *O. sativa japonica* (br1), *O. rufipogon* (br2), *O. sativa* subspecies *indica* and *O. nivara* (br3), *O. glaberrima* and *O. barthii* (br4), *O. glumaepatula* (br5), *O. meridionalis* (br6), *O. punctata* (br7), *O. brachyantha* (br8), and *L. perrieri* (br9; Fig. 1c).

In this study, 97% of rice de novo genes are within 1 million years (br1 to br5, 169/175). To make de novo genes and gene duplicates comparable in timescale, most analyses were based on genes with ages within 2.41 million years (br1 to br6). A previous study proposed “homology detection failure” as an alternative explanation for young genes (Weisman et al. 2020), which was a simplified null model assuming a constant evolutionary rate of protein-coding genes across species and no genetic novelty. This null model predicted that 85 “young genes” in five yeast species could be due to “homology detection failure” over 20 million years of evolution (155 × 55% = 85; Weisman et al. 2020). Considering the mutation rates of yeast and rice, which are 1.7 × 10^−7^ and 6.5 × 10^−9^ substitutions per site per generation, respectively (Liu et al. 2017; Gou et al. 2019), the number of rice genes under this null model within 2.4 million years could be very low (0.16). Together, our synteny-based approach and the extremely short timescale can provide reliable resolution for new gene identification and comparative study.

**Fig. 2.**
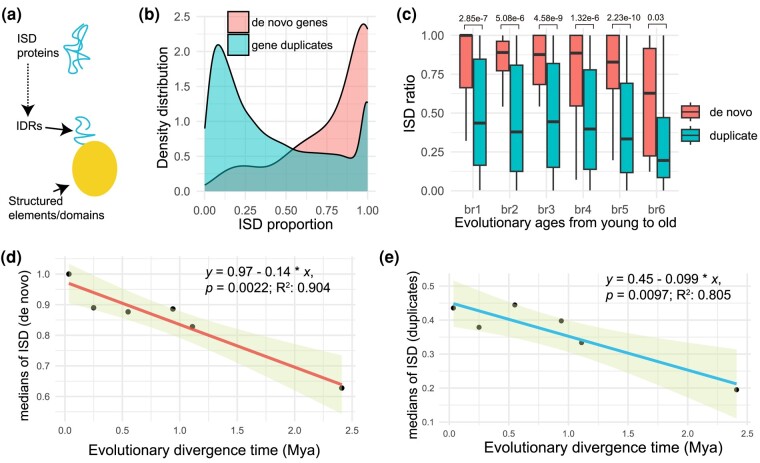
Analysis of ISD in de novo genes and gene duplicates. a) Illustration of an ISD protein highlighting the IDRs. b) Distribution comparison of IDRs’ fractions in de novo genes vs. gene duplicates. c) Boxplot representation of IDRs fractions (ISD ratio) in proteins for de novo genes and gene duplicates, categorized by evolutionary age from young to old (*x* axis). Differences are assessed using the Wilcoxon test, with the *P* value indicated above each comparison. d) A significant linear regression analysis showing the relationship between the median ISD fractions and the evolutionary ages of de novo genes. The 95% confidence interval is represented by the shaded area. e) Similar linear regression analysis for gene duplicates (br1 to br6), with the median ISD fractions plotted against evolutionary ages. The shaded area indicates the 95% confidence interval. The linear regression formula, *P* value, and adjusted *R*^2^ values are displayed at the top right corner.

Using an alignment-free tool Metapredict, a fast deep learning method that utilizes a bidirectional recurrent neural network trained on known disordered proteomes (Emenecker et al. 2021), our analysis characterized the ISD and its statistical distribution of de novo genes (supplementary table S1, Supplementary Material online). We discovered that 37.57% (68 out of 181) of de novo proteins exhibit complete ISD, characterized by being composed entirely of IDRs (Fig. 2a). Notably, this proportion far surpasses the 9.77% of complete ISD proteins in gene duplicates from age groups br1 to br6 (823 out of 8427). The overall distributions of ISD ratio (the ratio of sequence as IDRs) further showed that de novo genes are strikingly different from gene duplicates in terms of both median value (0.88 vs. 0.31) and distribution peak (0.97 vs. 0.08; Fig. 2b). Interestingly, we found that de novo genes gradually reduce in fractions of IDRs (regions of ISD), suggesting the reduction of disorder over evolutionary time (Fig. 2c). Specifically, the fractions of IDRs in de novo proteins have decreased by about 40% from the most recent branch (br1) to the oldest one (br6). In addition, de novo genes demonstrated a consistent pattern of higher proportions of IDRs than gene duplicates at all evolutionary stages within ∼1 to 2 million years (br1 to br6), despite a reduced difference between them at the oldest stage br6 (Fig. 2c). This pattern suggests that ISD levels in proteins are not stagnant over evolutionary time in rice. Statistically, a significant linear trend emerged: the proportions of IDRs in de novo proteins decreased by about 14% per protein per million years (Fig. 2c; *P* = 0.0022, adjusted *R*^2^ = 0.904). We also used AUCpreD (Wang et al. 2016) to identify ISD of de novo genes with default parameters and found patterns consistent with those obtained from Metapredict (supplementary fig. S1a, Supplementary Material online). The proportion of disordered regions was found to decrease by 14% per million years over evolutionary time, a rate identical to that reported by Metapredict (supplementary fig. S1c, Supplementary Material online). This consistency suggests that the observed evolutionary trends of ISD are unlikely to be artifacts of computational errors from specific method. Using the median ISD ratio of gene duplicates (0.31) based on Metapredict as a benchmark, and guided by this linear model, de novo proteins would require approximately 4.7 million years to attain the median disorder level observed in gene duplicates.

For gene duplicates, we found that 19.57% (1,818 out of 9,289) of proteins encoded by younger duplicates (Branches br2 to br5, ∼1 Mya) are categorized as ISD proteins (using 100% of residues in IDRs as the threshold). This rate is 8.4 times higher than that observed in older duplicates from Stages br6 to br9 (2.32%, 570 out of 24,620; supplementary table S1, Supplementary Material online). For the *O. sativa Japonica*-specific duplicates (br1), we divided the duplicates into two groups: young-parent duplicates and old-parent duplicates, based on the evolutionary epochs from which their parent gene emerged (br2 to br5 as young parent vs. br6 to br9 as old parent). Our analysis revealed a significantly higher fraction of ISD proteins in young-parent duplicates compared with old-parent duplicates (58.60%, 53 out of 215 vs. 32.14%, 26 out of 252; odds ratio 2.38, 95% confidence interval: 1.44 to 3.95, *P* = 0.0007; supplementary table S1 and fig. S1b, Supplementary Material online). This finding suggests that gene duplicates may inherit structural properties from their parental genes. When we analyzed br1 duplicated genes without separating them, we discovered that 16.70% (78 out of 467) of the br1 duplicates are ISD proteins, a proportion that remains higher than that of ISD proteins in the br2 age group, which stands at 13.50% (96 out of 711, supplementary table S1, Supplementary Material online).

In our comparative analysis of the evolutionary rate of ISD fractions between de novo genes and gene duplicates across Branches br1 to br6 (Fig. 2d and e), we uncovered a notable trend. De novo genes exhibit a 4% faster rate of disorder decay per million years than gene duplicates on average, with respective slopes of 0.14 vs. 0.099. This accelerated rate in de novo genes may stem from their absence of the intrinsic heritage effect, which in turn could contribute to their heightened evolvability in regard to ISD compared with gene duplicates.

### Rapid Evolution of Structural Elements in De Novo Proteins

**Fig. 3.**
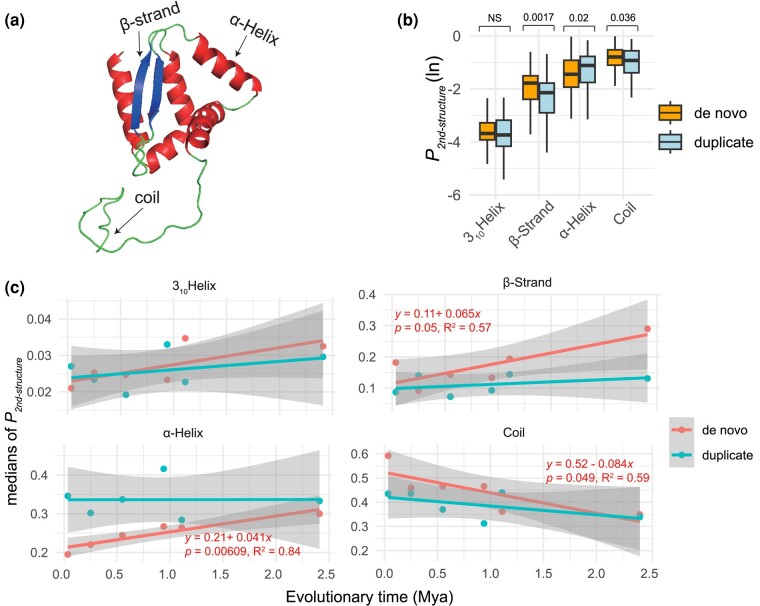
The length proportions of structural elements (noted as *P*_2nd-structure_, transformed using the natural logarithm), including unstructured (coil) and structured segments (3_10_ helix, α helix, and β strand) and their correlations with gene ages. a) An example of basic elements of protein structure. The visualization is based on the ranked_0 result of AlphaFold2 for de novo gene Osjap03g04570. b) The distributions and comparisons for length proportions of coil and other structured region segments (α helix, 3_10_ helix, and β strand). The comparisons are based on Wilcox test, and *P* values are shown above boxplots. c) The linear regression of *P*_2nd-structure_ for de novo genes against evolutionary time. The linear statistical summaries and formulas are indicated in red for de novo genes. The regression statistics of gene duplicates are not shown due to insignificant *P* values for all elements.

In protein structure, α helices and β strands are typically amphipathic and thus can enable the tertiary folding of hydrophilic surfaces and hydrophobic cores (Fersht 1999). The α helices (and other helices like 3_10_ helices) and β strands (which form β sheets) are considered structured due to their specific, stable hydrogen-bonding patterns, while random coil regions lack such regular structure and are more flexible and disordered (Craveur et al. 2015;  Fig. 3a). We conducted a comparative analysis of these structural elements for de novo genes and gene duplicates, focusing on relative proportions of these structural elements within protein sequences over evolutionary time. We predicted protein 3D structures with AlphaFold2 (supplementary figs. S2 to S7, Supplementary Material online for the structures of de novo genes originated from Branches 1 to 6) and decoded the structural elements with STRIDE (Heinig and Frishman 2004; Jumper et al. 2021). We finally measured the lengths and proportions of these structural elements (*P*_coil_ for coil, *P*_helix_ for α helices, *P*_310helix_ for 3_10_ helices, and *P*_strand_ for β strands). Our analysis revealed that median proportion values are highest in unstructured coils (40% to 47%) and followed by α helices (23% to 30%), β strands (13% to 15%), and 3_10_ helixes (2.7% to 2.8%) for de novo genes and gene duplicates (supplementary table S2, Supplementary Material online).

Overall, the *P*_coil_, *P*_helix_, and *P*_strand_ are significantly different between de novo genes and gene duplicates, while no significant difference was found for 3_10_ helixes (Fig. 3b). In de novo genes, our analysis revealed a strong negative linear correlation between median of *P*_coil_ and gene age, alongside significant positive linear correlations between both median of *P*_helix_  *and P*_strand_ and gene age (Fig. 3c). These correlations suggest a faster evolutionary rate in the structural elements of de novo genes over time, marked by an increase in novel structures and a decrease in unstructured coil segments. Specifically, α helix and β strand grow with rates of 4.1% and 6.5% per protein per million years, respectively, while coil decreases with a rate of 8.4% per protein per million years (Fig. 3c). In contrast, such correlations are not significant for the linear model in gene duplicates (Fig. 3c). To understand the pattern of duplicated proteins with higher sample size, we downloaded all predictions for rice protein structures from AlphaFold2 database (https://alphafold.ebi.ac.uk/; v4). Following a strict conversion between different genome annotations (see Materials and Methods), we obtained 9,433 duplicated proteins with predicted structures and decoded the secondary structure with STRIDE (Heinig and Frishman 2004; Jumper et al. 2021). We observed that the linear model was inadequate for describing the changes in the proportions of secondary structural elements in proteins that have undergone duplication, when looking across evolutionary timescales expressed in millions of years (Mya). However, we found that significant nonlinear models with logarithmic time unit could fit the data (supplementary fig. S1d, Supplementary Material online). We observed that, over the logarithmic timescale, the fractions of β strands significantly increase (*P* = 0.02 and *R*^2^ = 0.72), while those of coil and isolated bridge significantly decrease (*P* = 0.013 and *R*^2^ = 0.77 for bridge; *P* = 0.0001 and *R*^2^ = 0.93 for coil). These patterns suggest that de novo proteins and duplicated proteins have different evolutionary rates of secondary structure elements, although the overall qualitative trends are similar with a decrease in disordered regions and an increase in structured regions over time. The quantitative difference between predicted ISD and secondary structure elements is consistent with the conditional folding of ISD (Alderson et al. 2023).

### The Properties of Amino Acids in De Novo Genes Are Consistent with the Structural Changes

**Fig. 4.**
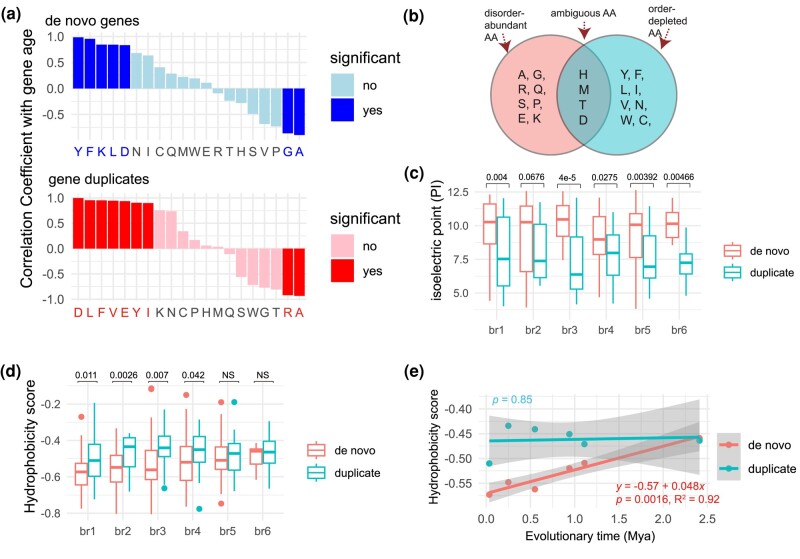
The correlation coefficient between compositions of amino acids and gene ages (Mya). a) The Pearson correlation coefficients (*r*) between amino acid fractions (medians) and their gene ages (Mya; supplementary table S3, Supplementary Material online). “Yes” and “no” indicate significant and nonsignificant *P* values, respectively. b) The classifications of amino acids (AA): disorder-promoting AA, order-promoting AA, ambiguous AA, based on a previous report (Dunker et al. 2001). c) The comparisons of isoelectric point between duplicates and de novo genes across six branches. d) The comparisons of hydrophobicity scores between duplicates and de novo genes across six branches. The larger values represent higher hydrophobicity. e) The linear regression of median hydrophobicity scores against evolutionary times. Statistical summaries are shown near regression lines with *P* values, adjusted *R*^2^ value, and formula. Comparisons are based on the single-tailed Wilcoxon rank-sum test.

The observed patterns for IDRs, random coils, and structured elements (α helices and β strands) in de novo proteins necessitate a more comprehensive analysis of amino acid composition to further understand de novo gene evolution. To understand whether the compositional fractions of some amino acids could be related to gene ages, for each amino acid, we assessed the correlation between median values of fractions and evolutionary ages (Fig. 4a). We also compared amino acid compositions and their correlations with gene ages between de novo genes and gene duplicates (Table 1 and supplementary fig. S8, Supplementary Material online).

**Table 1:** The comparisons between proteins of de novo genes and duplicated genes

Amino acid	Polarity	Codon degeneracy	Codons	Charge	Volume	Other important properties	Abundance in de novo genes	*P* value
G	Nonpolar	4	GGT, GGC, GGA, and GGG	Neutral	Small	Hydrophobic core	Higher	3.25E^−05^
*P*	Polar	4	CCT, CCC, CCA, and CCG	Neutral	Small	Proline kinks	Higher	2.56E^−05^
R	Polar	6	CGT, CGC, CGA, CGG, AGA, and AGG	Positive	Large	…	Higher	1.59E^−13^
D	Polar	2	GAT and GAC	Negative	Small	…	Lower	2.71E^−16^
E	Polar	2	GAA and GAG	Negative	Medium	…	Lower	1.23E^−03^
F	Nonpolar	2	TTT and TTC	Neutral	Large	Aromatic ring	Lower	3.27E^−08^
I	Nonpolar	3	ATT, ATC, and ATA	Neutral	Large	Hydrophobic core	Lower	1.17E^−07^
L	Nonpolar	6	TTA, TTG, CTT, CTC, CTA, and CTG	Neutral	Large	Hydrophobic core	Lower	1.67E^−09^
N	Polar	2	AAT and AAC	Neutral	Small	Amide group	Lower	7.17E^−04^
V	Nonpolar	4	GTT, GTC, GTA, and GTG	Neutral	Medium	Hydrophobic core	Lower	3.65E^−10^
Y	Polar	2	TAT and TAC	Neutral	Large	Hydroxyl group	Lower	3.53E^−09^

The *P* values are statistical differences between de novo genes and gene duplicates based on the Wilcox test (significance threshold 0.0025 is adjusted by the multiple test). The field of “codon degeneracy” indicates the numbers of codons for the corresponding amino acids.

Among all amino acids, the average fractions of alanine (A) and glycine (G) exhibited significant negative correlations with ages of de novo genes (Fig. 4a and supplementary table S3, Supplementary Material online). This result suggests that a disorder-promoting tendency of alanine and glycine could promote the higher ISD and fractions of unstructured coils in young de novo genes (Fig. 4b;  Dunker et al. 2001; Uversky 2013). In gene duplicates, alanine (A) and arginine (R) were the two amino acids whose fractions significantly negatively correlated with gene ages (Fig. 4a). Arginine (R) has lower disorder propensity than glycine (G; Uversky 2013). The difference is consistent with our finding of a higher degree of ISD in de novo genes compared with gene duplicates.

Tyrosine (Y), phenylalanine (F), lysine (K), and leucine (L) exhibited significant positive correlations with the ages of de novo genes (Fig. 4a and supplementary table S3, Supplementary Material online), suggesting their roles in the rapid structural evolution of these genes. Notably, 75% (3 out of 4: Y, F, and L) of these amino acids are hydrophobic and order promoting, with low disorder propensities (Dunker et al. 2001; Tompa 2002; Uversky 2013). The lysine (K) is positively charged, which could favor salt bridge to interact with negatively charged amino acids or interactions with DNA or RNA (Couso and Patraquim 2017). Comparative analysis revealed that de novo proteins collectively have significantly higher fractions of glycine (G), proline (P), and arginine (R) than gene duplicates (supplementary fig. S8, Supplementary Material online). These amino acids are characterized by high codon degeneracy and encoded by GC-rich codons (Table 1), which is consistent with high GC content in rice de novo genes (Zhang et al. 2019). Previous studies conducted on yeast, flies, and mammals suggest that new proteins are usually positively charged (Blevins et al. 2021; Papadopoulos et al. 2021; Montañés et al. 2023). We found that de novo proteins are significantly higher in fraction of positively charged amino acid residue R (arginine) and lower in fractions of negatively charged glutamate residue (E) and hydrophobic amino acid residue (F; Table 1).

### De Novo Proteins: Lighter, Positively Charged, and Increasingly Hydrophobic Over Time

Despite these findings, the extent to which this characteristic is pervasive among proteins of varying evolutionary ages remains uncertain. We compared several physiochemical properties, including protein charge, molecular weight, and hydrophobicity, between proteins from de novo genes and gene duplicates across evolutionary stages. By evaluating isoelectric point, we found that de novo proteins exhibit significantly higher positive charges than gene duplicates in all evolutionary age groups except br2 (*P* < 0.05; Fig. 4c). Among 20 amino acids, there are three basic (K, H, and R) and two acidic (D and E) amino acids. We found a significant positive correlation between the fractions of the aspartic acid (D) and gene ages (Fig. 4a), in addition to significantly lower fractions of the aspartic acid (D) at the youngest five stages (br1 to br5; supplementary fig. S8a, Supplementary Material online), consistent with a previously reported depletion of this amino acid in younger de novo proteins in flies (Montañés et al. 2023). We further found significantly higher fractions of arginine (R) in de novo proteins than in duplicated proteins at the youngest five stages (br1 to br5; supplementary fig. S8a, Supplementary Material online). Together, the younger de novo proteins are higher in basic amino acid (arginine R) while lower in acidic amino acid (aspartic acid D) at five age groups, which could explain the pattern of positive charge in de novo genes (Fig. 4c). Moreover, compared with duplicated proteins, de novo proteins displayed significantly shorter protein lengths at all evolutionary age groups and significantly lower molecular weights (Da) at five age groups (br2 to br6; supplementary fig. S8c and d, Supplementary Material online).

De novo proteins also showed significantly higher hydrophobicity scores than duplicated proteins at the first four evolutionary stages within 0.94 million years (br1 to br4; Fig. 4d), and no significant difference was found at br5 (∼1 Mya) and br6 (∼2 Mya; Fig. 4d). Moreover, only in de novo proteins, we detected a significant increasing trend of hydrophobicity score over time with the growth rate of 4.8% per protein per million years (Fig. 4e). Due to the dominant role of hydrophobic interactions in driving protein folding, the growth of hydrophobicity over time strongly supports the faster evolution of folding in de novo proteins than in proteins from gene duplication (Fig. 4e), which is also consistent with the patterns of secondary structure elements (Fig. 3c).

### Protein Complex Interaction Could Facilitate the Structural Evolution of De Novo Protein

We computationally generated and analyzed the tertiary folding or 3D structure for all de novo genes and a random selection of duplicated genes (30 genes per age group; Materials and Methods). The pLDDT score provides information for modeling confidence, disorder levels, and structural variability (Saldaño et al. 2022; Wilson et al. 2022). We compared pLDDT scores between de novo genes and gene duplicates (supplementary fig. S9a, Supplementary Material online). The median pLDDT scores were consistently higher in gene duplicates than in de novo genes, suggesting a greater confidence in the modeling predictions for the tertiary structures of duplicated proteins (supplementary fig. S9a, Supplementary Material online). This pattern could also reflect our findings of higher levels of ISD in de novo genes (Fig. 2c), considering the correlation between pLDDT and disorder (Saldaño et al. 2022; Wilson et al. 2022; Tesei et al. 2024). To understand whether the predicted structures of de novo proteins could be randomly modeled, we estimated pairwise TM scores for all models of AlphaFold2. A TM score exceeding 0.5 suggests a similar fold, while a TM score below 0.17 signals that structural likeness is nearly random (Mukherjee and Zhang 2009; Xu and Zhang 2010). We found only one de novo protein (Osjap01g35740, br4) with median TM score less than 0.17 while 14.29% of de novo proteins (25 out 175) with median TM score over 0.5 (supplementary table S4, Supplementary Material online). In addition, all median TM scores across age groups of de novo proteins are over 0.17, although these values are significantly lower than those of duplicated proteins (supplementary fig. S9b, Supplementary Material online). These results suggest that the structures for most of de novo proteins were not randomly modeled in AlphaFold2.

We further categorized proteins into three distinct groups based on their folding characteristics, as indicated by pLDDT (supplementary table S4, Supplementary Material online; the three groups with pLDDT values 0 to <0.7, ≥0.7 to <0.9, and ≥0.9 to 1.0, as expressed as a fraction of the maximum value). We found that 3.43% of de novo genes (6 out of 175) have the high pLDDT values in at least one element over ten continuous amino acids (pLDDT ≥ 0.9) and 17.14% of de novo genes (30 out of 175) have elements with confident scores (pLDDT ≥ 0.7; supplementary table S4, Supplementary Material online). Among these predicted genes, only six genes have two structural elements while the rest of them (24) have at most one structural element (α helix or β sheet), consistent with previous observations of limited folding in de novo gene-encoded proteins in other species (Peng and Zhao 2024). It is notable that low pLDDT does not always correlate with disorder (Middendorf and Eicholt 2024). Filtering by pLDDT could filter out folded structures predicted with low confidence considering the case of conditional folding (Alderson et al. 2023), thereby leading to a potentially conservative estimation in our analysis.

**Fig. 5.**
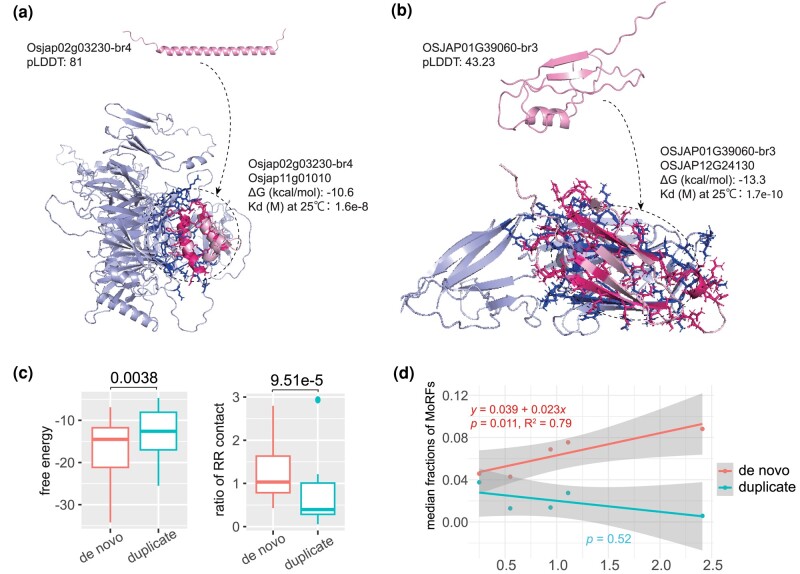
The visualization and statistics of structures for proteins and complexes. a) The 3D structures of Osjap02g03230 and its protein complex. pLDDT indicates average value for all four models, showing a well-folded example (pLDDT expressed as a fraction value from 0 to 1.00). The dotted circle shows the binding state of this de novo protein. b) The 3D structures of OSJAP01G39060 and its protein complex. pLDDT indicates average value for all four models, representing a not well-folded example. c) The comparisons of numbers of RR pairs and Gibbs free energies (kcal/mol) from results of protein complexes (the model ranked_0) with AlphaFold2-multimer between de novo proteins and duplicates. All comparisons are estimated with the single-tailed Wilcoxon test (*P* values shown above). d) The regression of linear model between median MoRF fractions and evolutionary years (Mya). The statistical summaries of linear model are listed for the two types of genes (de novo genes and duplicates).

Most proteins function through interactions with other proteins, a process that can induce conformational changes, particularly in disordered proteins (Zhang et al. 2013; Tsaban et al. 2022). To explore the likelihood of disorder-to-order transitions during these interactions over time, we assessed the length proportions of MoRFs, which are prone to conformational changes during protein–protein contact. We found that MoRF fractions are consistently higher in proteins from de novo genes than duplicated genes, although statistical significances were only found in older evolutionary ages (*P* < 0.05, br3 to br6; supplementary fig. S9c, Supplementary Material online). In de novo genes, we observed a significant linear increase in the median MoRF fractions over evolutionary time, growing at 2.3% per protein per million years (br2 to br6; Fig. 5d). These findings suggest that de novo genes could evolve de novo MoRFs for molecular recognition during binding.

Using gene coexpression correlation analysis of RNA-seq data (supplementary table S5, Supplementary Material online) and based on several criteria including disorder levels (ISD proportions < 5%) and high correlation coefficients (>0.8; see Materials and Methods), we identified 30 pairs of potential protein–protein interactions involving de novo proteins (supplementary table S6 and fig. S10, Supplementary Material online). We also used the same criteria and randomly chose 30 pairs of coexpressed gene duplicates for comparison (supplementary table S6 and fig. S11, Supplementary Material online). Based on resulting models of the AlphaFold2-multimer, we compared structural consistency among modeled complexes between de novo and duplicated proteins using pairwise TM score (Mukherjee and Zhang 2009). Due to lower sample size of complexes, we only compared the overall difference of TM scores between de novo and duplicated protein complexes. We found no significant difference of TM scores between the two groups (supplementary fig. S9d, Supplementary Material online), suggesting a comparable modeling stability for complexes of de novo proteins and duplicated proteins obtained from AlphaFold2-multimer. In addition, we found all de novo protein complexes with TM scores over 0.17 and only five de novo protein complexes with median TM scores lower than 0.5 (supplementary table S7, Supplementary Material online). These distributions indicate that most of de novo protein complexes have similar folds for all refined models from AlphaFold2-multimer. The different patterns of TM scores between single protein prediction and complex prediction also suggest that the low-confidence modeling of some de novo proteins may not influence the modeling confidence of protein complexes (supplementary fig. S9, Supplementary Material online). This finding is consistent with a previous evaluation of AlphaFold-multimer that highly confident structures could be obtained for some proteins without homology to any existing structures (Zhu et al. 2023).

We observed the possibility of de novo protein complex formation and potential conformational change upon protein–protein interaction (Bryant et al. 2022; Evans et al. 2022; Tsaban et al. 2022). In one instance, de novo gene Osjap02g03230, which exhibited a highly confident folding structure with a single α helix, had a predicted conformational change to two α helices upon binding to its potential protein partner Osjap11g01010, a geranylgeranyl transferase Type-2 subunit beta-like protein, with very low free energy (Δ*G* = −10.6; Fig. 5a). The protein complex prediction based on AlphaFold2-multimer indicated a conformational change into a “helix-turn-helix” motif upon binding. Δ*G* values are generally in the range of −5 to −10 kcal/mol for biologically relevant interactions (Yugandhar and Gromiha 2014). Thus, the estimate of Gibbs free energy (Δ*G*) suggests a strong biologically relevant binding affinity for this protein complex based on the reported cutoff (Δ*G* around −10; Yugandhar and Gromiha 2014; Nikam et al. 2023). Another de novo gene, OSJAP01G39060, showed a stronger binding affinity, as indicated by low Δ*G* and *Kd* values (Δ*G* = −13.3; Fig. 5b). Moreover, two more β strands were observed in this protein complex, supporting the potential structural and conformational change upon binding. These two groups of Δ*G* and *Kd* values indicate that the binding processes could be spontaneous and stable for de novo proteins. Future comparative studies with randomly generated sequences would yield more detailed insights into the protein binding process.

Using the Gibbs free energy (Δ*G*) as the indicator of protein–protein binding affinity, we found that de novo proteins have significantly stronger binding affinities with their partners than proteins from gene duplicates (median −16.67 vs. −13.08, single-tailed Wilcoxon rank-sum test, *P* = 0.021; Fig. 5c). This observation is also consistent with our finding of significantly more RR contacts in de novo protein complexes than in those from gene duplicates (median 183 vs. 125, single-tailed Wilcoxon rank-sum test, *P* = 0.0038). On average, RR pairs were estimated to be 4.71% more in de novo protein complexes than in protein complexes of duplicated proteins (12.22% vs. 7.51%; supplementary table S6, Supplementary Material online). By normalizing with protein length, we still found significantly higher RR contacts per amino acids in de novo proteins than in duplicated proteins (Fig. 5c; Wilcoxon rank-sum test, *P* = 9.51E^−5^). These results strongly suggest that the disordered and flexible nature of de novo proteins could facilitate strong binding between proteins. Notably, among all 30 pairs of de novo protein interactions studied (supplementary table S6 and fig. S10, Supplementary Material online), we revealed only 17% of potential protein complexes (5 out of 30) with Δ*G* values higher than −10 kca/mol, suggesting that most of de novo genes (83%) can form highly compact and high-affinity complexes with low free energy (supplementary table S6b, Supplementary Material online). Together, our results suggest that de novo proteins could form stable complexes with biological relevant binding and may even undergo significant conformational changes.

## Discussion

### De Novo Proteins Gradually Evolve in Structural Complexity More Quickly Than Gene Duplicates, Forming Protein Complex with Previously Existing Proteins

Both de novo genes and gene duplicates are important raw materials for evolutionary innovation (Long et al. 2013), with similar persistence rates in deep evolutionary lineages (Montañés et al. 2023). As a predominant part of protein-coding genes in genomes, gene duplicates have been modeled to have multiple possible consequences of functional evolution, including neofunctionalization that creates novel functions (Ohno 1970; Birchler and Yang 2022). However, the possibility of origination and functionalization of de novo genes was long dismissed (Jacob 1977; Mayr 1982). Nevertheless, recent studies have provided substantial evidence for the importance of de novo genes in origins of functional novelties (Cai et al. 2008; Suenaga et al. 2014; Gubala et al. 2017; Xie et al. 2019; Zhuang and Cheng 2021; Weisman 2022; An et al. 2023; Chen et al. 2023; Qi et al. 2023), although it is unknown whether the de novo genes and duplicates are evolutionarily persistent in comparable rates. The structural analysis in this study reveals fresh insights into structural reasons by which these de novo genes evolved to acquire new protein functions.

The structure–function relationship in structural biology suggests that a protein's primary sequence dictates its tertiary conformation, which in turn defines protein functions (Anfinsen and Haber 1961). This underscores the importance of investigating the structural evolution of proteins, particularly in the case of de novo proteins. With cutting-edge computational tools now available, researchers have begun on detailed case studies to elucidate the foldability and inherent structure of de novo genes (Bungard et al. 2017; Bornberg-Bauer et al. 2021; Lange et al. 2021). Previous studies reported little change in structure over millions of years (Peng and Zhao 2024; Lange et al. 2021). Our analyses revealed that the de novo genes evolved gradually in terms of their structural complexity in a short timescale. We showed that de novo genes in rice structurally evolved faster than gene duplicates, suggesting the initial structures of new genes created from noncoding sequences are more flexible to evolve toward different functions. In fact, the strong positive selection observed in the de novo genes that favor enabler mutations is in line with the observation of their rapid structural evolution. Furthermore, we found that the de novo proteins participated in a protein complex with a structural role distinct from its structure as a monomer, by interacting with previously existing proteins encoded by older genes.

### De Novo Proteins Initially Exhibit High Disorder But Rapidly Evolve Toward Structured Forms

By comparing our previously identified de novo genes with gene duplicates across well-ordered evolutionary timescales (Zhang et al. 2019), we measured quantitively that the median proportion of IDRs is 88%. This result indicates disorder as a predominant characteristic for these proteins over a period of 1 to 2 million years. The structural versatility of IDRs could confer special molecular advantages for de novo proteins, allowing them to adapt to almost every cellular compartment and perform various functions, including transcription, nuclear transport, RNA binding, signaling, and cell division (Holehouse and Kragelund 2023). For instance, numerous RNA-binding proteins and transcription factors, which are known to bind nucleic acids and mediate protein–RNA or protein–DNA interactions, contain IDRs (Brodsky et al. 2020). Another significant example is the IDRs found in eukaryotic histone tails and RNA polymerase II C-terminal domain, which undergo posttranslational modifications essential for gene expression regulation throughout development (Jiao et al. 2020).

We also found a rapid evolution of their protein structures compared with proteins from gene duplicates within the time frame of 1 to 2 million years. This rapid evolution is characterized by a decrease in the proportion of unstructured regions (random coils) and an increase in structured regions, such as α helices and β strands. We also detected signals of MoRFs and their growing pattern over time. Previous studies have shown mixed results regarding IDRs in proteins across different species. Higher levels of ISD in younger proteins were found in humans, mice, and flies (Wilson et al. 2017; Peng and Zhao 2024). In contrast, Dowling et al. (2020) observed no significant changes in ISD over time in human de novo open reading frames, indicating a stable pattern of intrinsic disorder across evolutionary timescales (Dowling et al. 2020). Our study quantitively measured the evolutionary rate for structural changes of de novo proteins at a finer scale. We found that, despite strikingly higher proportions of IDRs for de novo proteins, the disorder decay rate is at 14% per protein per million years, which is faster than that in duplicated proteins with 9.9% per protein per million years.

We further observed distinct evolutionary patterns in the basic elements of protein folding. Specifically, we estimated a decrease in random coils at a rate of 8.4% per protein per million years, which suggests a reduction in less structured regions where weaker interactions like Van der Waals forces are predominant. Conversely, there was an increase in α helices and β strands at rates of 4.1% and 6.5% per million years, respectively. This increase indicates a shift toward more structured and stable configurations, typically stabilized by hydrogen bonding within the protein's backbone. The growth in α helices and β strands suggests an evolutionary trend toward more hydrogen bond-rich and intricately folded structures, possibly reflecting an increased need for functional specificity and molecular stability. We revealed a pattern of increasing hydrophobicity in de novo proteins at 4.8% per protein per million years, suggesting an enhanced role of hydrophobic interactions in stabilizing the protein's tertiary structure and promoting the interior packing of hydrophobic side chains.

### Multiple Features of De Novo Proteins Could Promote the Formation of Protein Complex

**Fig. 6.**
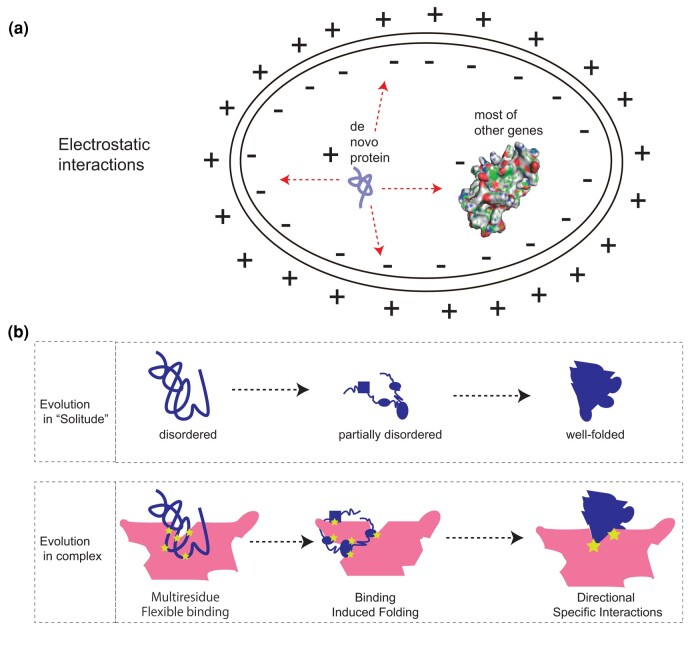
The schematic illustration for molecular diffusion and structural evolution of de novo proteins. a) The schematic molecular diffusion and movement showing differences in diffusion speed based on protein charges and molecular weight differences between de novo genes and duplicates (also see supplementary fig. S8c, Supplementary Material online for molecular weight differences). The “+” indicates the general positive charges in de novo proteins and outside of the cell membrane. The “−” indicates the more negatively charged proteins from duplicates and the inner side of the cell membrane. The size difference indicates the general pattern of significantly less molecular weight in de novo genes than in gene duplicates. b) Two models of protein folding evolution for de novo protein: the EIS model and the EIC model.

Our analyses indicated several unique physiochemical features of de novo proteins compared with proteins of gene duplicates, which could promote the interactions between de novo proteins and other proteins. Although previous findings in other species have revealed significantly higher positive charges in de novo proteins than other genes (Blevins et al. 2021; Papadopoulos et al. 2021; Montañés et al. 2023), it was unknown whether that is general for all evolutionary ages in rice. Our analyses revealed the general patterns of higher positive charges for de novo proteins than duplicated ones in age groups where divergence occurred ∼2 million years before the present or less. We also revealed the generally smaller molecular weights of de novo proteins than proteins of gene duplicates. Proteins with greater opposite charges could promote stable binding to form complexes (Hazra and Levy 2022). Thus, the tiny and attractive features in terms of weight and charge may suggest a faster-binding scenario for de novo proteins, where the nascent de novo proteins could have relatively higher diffusion speed to be attracted to the negatively charged compartments or larger molecules (Fig. 6a). Generally, larger negatively charged proteins tend to offer greater collision cross sections for interactions, while smaller positively charged proteins, with their faster diffusion, are more prone to molecular collisions (Xu et al. 2013; Morris et al. 2022). Therefore, our results suggest that de novo proteins, exhibiting generally positive charge and smaller size, may have a higher diffusion potential, increasing their likelihood of interacting with larger, negatively charged proteins or cellular structures.

Our 3D structural analyses on de novo proteins and complexes revealed contrasting patterns between the isolated protein structure and protein complex. Consistent with the expectation based on high levels of ISD in de novo proteins and findings in other species (Peng and Zhao 2024), we found that the tertiary structures of de novo genes in isolation are simple with limited number of structural elements and not well folded in general. Only a tiny percent (3.43%) of de novo protein had confidently modeled folding structures based on AlphaFold2. This general feature could reflect the nature of disorder propensities of de novo proteins. We found that TM scores are significantly lower for models of predicted structures of de novo proteins than for those of duplicated proteins. Despite this difference, TM scores also revealed that the predicted structures of de novo proteins could not be randomly modeled in general. Surprisingly, however, AlphaFold2-multimer analyses suggested that most de novo protein complexes (83%) have high binding affinities (Gibbs free energy < −10), despite the disordered nature of de novo proteins in isolation. TM scores for complexes revealed no significant difference between de novo protein complexes and duplicated protein complexes with medians over 0.5, supporting similar folds among predicted complex structures for de novo protein complexes. The comparison between protein monomer and complex demonstrated potential conformational changes for de novo proteins upon interaction. The RR contacts per amino acid are higher in the de novo protein complex than in the duplicated protein complex. Probably constrained by the rigid bodies of well-folded conserved proteins, previous study has found that interfaces of protein–protein interaction are generally controlled by a small and complementary set of contact residues that maintains most of the binding affinity (Clackson and Wells 1995). Thus, our findings suggest that de novo protein complexes in both cases could be formed more easily than duplicated protein complex in general.

### Two Models for Structural Evolution of De Novo Proteins

A previous study has been suggested that de novo proteins could quickly interact with other proteins (Bornberg-Bauer et al. 2021). From observed structures and structural evolution of de novo proteins, we propose two complementary models to interpret the structural evolution of de novo proteins: the evolution in solitude (EIS) and the evolution in complex (EIC) with other proteins (Fig. 6b). The EIS model emphasizes the intuitive and isolated way of structural evolution step by step over evolutionary time from disordered to partially disordered and then to well folded. Our results have revealed a few distinguished features of de novo proteins, including high positive charges (Fig. 4c), small molecular weights (supplementary fig. S8c, Supplementary Material online), more RR contacts in complexes (Fig. 5c left), lower free energy in complexes (Fig. 5c right), and widespread strong binding for most of de novo proteins (>83%). These features are in support of the second model EIC that emphasizes the role of protein complex composed of de novo protein and well-folded protein in inducing the evolution of folding domains. The EIC model is also consistent with the previous findings that folding is not necessary for binding (Chebaro et al. 2015) and network hub proteins tend to be disordered (Haynes et al. 2006; Midic et al. 2009). In the EIC model, the formation of de novo protein complex could be instant and unspecific after protein emergence, much earlier than the formation of well-folded protein structure in isolation. The EIC model suggests that the tertiary structure evolution of de novo proteins could go through steps from the multiresidue binding (Fig. 5c), the binding-induced folding (Fig. 5a and b), and to potentially directional specific binding. The binding-induced folding might be a key mechanism facilitating the rapid decrease in disorder within de novo proteins, presenting an intriguing area for future research.

Overall, our study demonstrates that de novo genes can evolve rapidly in structural elements within a relatively short evolutionary timeframe. We estimated in this study that gene duplicates represent over 70% of rice protein-coding genes. Despite this abundance, de novo genes in general have faster evolutionary rate in structural changes, which highlight the importance of de novo gene emergence as a distinguished source of genetic innovation in organisms. The faster binding of de novo genes prior to their well-folded structures could be one of the mechanisms through which de novo genes are fixed in the population, evolve rapidly to acquire new functions, and integrate into existing biological networks by protein–protein interactions. Despite these intriguing patterns, we acknowledge that there could be some potential limitations for AlphaFold2-based prediction for de novo proteins (Aubel et al. 2023; Liu et al. 2023; Middendorf and Eicholt 2024). Future research in this area by incorporating random sequences, more complexes, and MD simulation could provide further insights into the mechanisms driving the rapid evolution of de novo genes and their impacts on the evolution of complex biological systems.

## Conclusion

Our research in rice indicates distinct patterns of rapid structural transformation in de novo genes over a relatively brief evolutionary timeframe of 1 to 2 million years. Additionally, we estimate that de novo proteins in rice require no longer than 5 million years to attain an intrinsic structural order comparable with that observed in gene duplicates. Exceptional characteristics of de novo genes, such as their low molecular weights, positive net charges, and strong binding affinities, and more RR contacts, likely drive their efficient diffusion and interactions with other proteins, which are essential for their evolution of biological functions. Hence, our findings highlight the unique mechanisms by which these continuously emerging de novo proteins in rice could rapidly form complexes in evolutionary history.

## Supplementary Material

evae107_Supplementary_Data

## Data Availability

All data and codes developed in this study are available at 10.5281/zenodo.10712836.
