# Supplementary material for: The Rapid Evolution of De Novo Proteins in Structure and Complex
Source: Genome Biol Evol. 2024 May 16;16(6):evae107. doi: 10.1093/gbe/evae107 (PMC11149777; doi:10.1093/gbe/evae107)
Supplement: evae107_Supplementary_Data [file evae107_supplementary_data.zip › Supplementary figures.pdf]

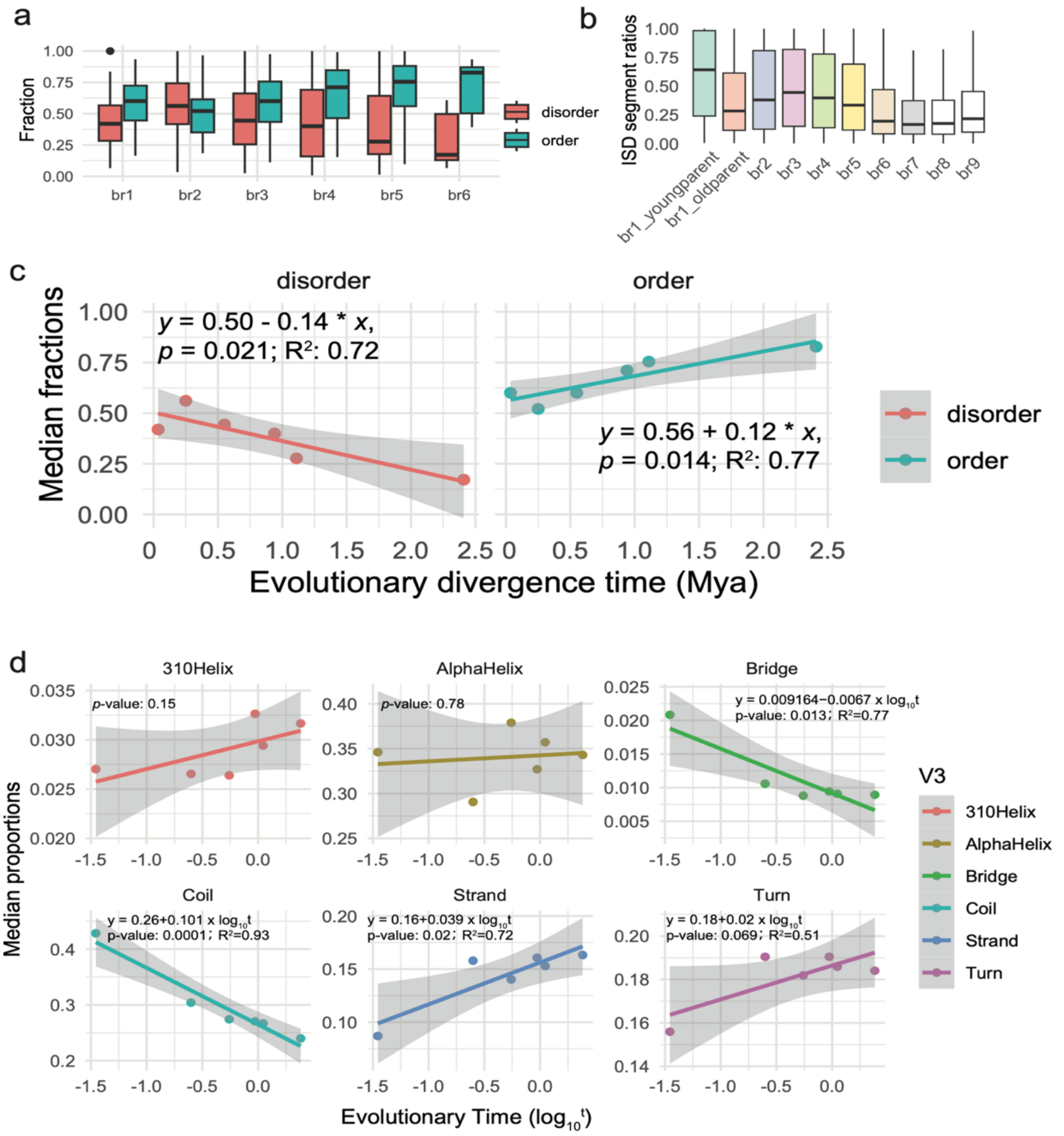

Supplementary figure 1. The replicate analysis of ISD with AUCpreD and ISD trend of gene duplicates. (a) The increasing trend of fraction of the ordered aminio acids and the decreasing trend of fraction of the disordered aminio acids with default parameter of AUCpreD. (b) The linear regression analysis to explore the relationship between the ages of genes (in million years ago, Mya) and the corresponding median values of the fractions of ordered and disordered amino acids for all de novo genes, based on default parameter of AUCpreD. (c) The fractions of intrinsic structure disordered regions for duplicated genes based on Metapredict across evolutionary ages from young to old. The br1_youngparent and br1_oldparent indicate young gene duplicates at br1 with parental genes from br2-5 and br6-9, respectively. (d) The regression analyses of $P_{2nd\text{-}structure}$ for duplicated proteins (based on Alphafold database structure) against evolutionary time (logarithmic unit). The statistical summaries and formulas are indicated.

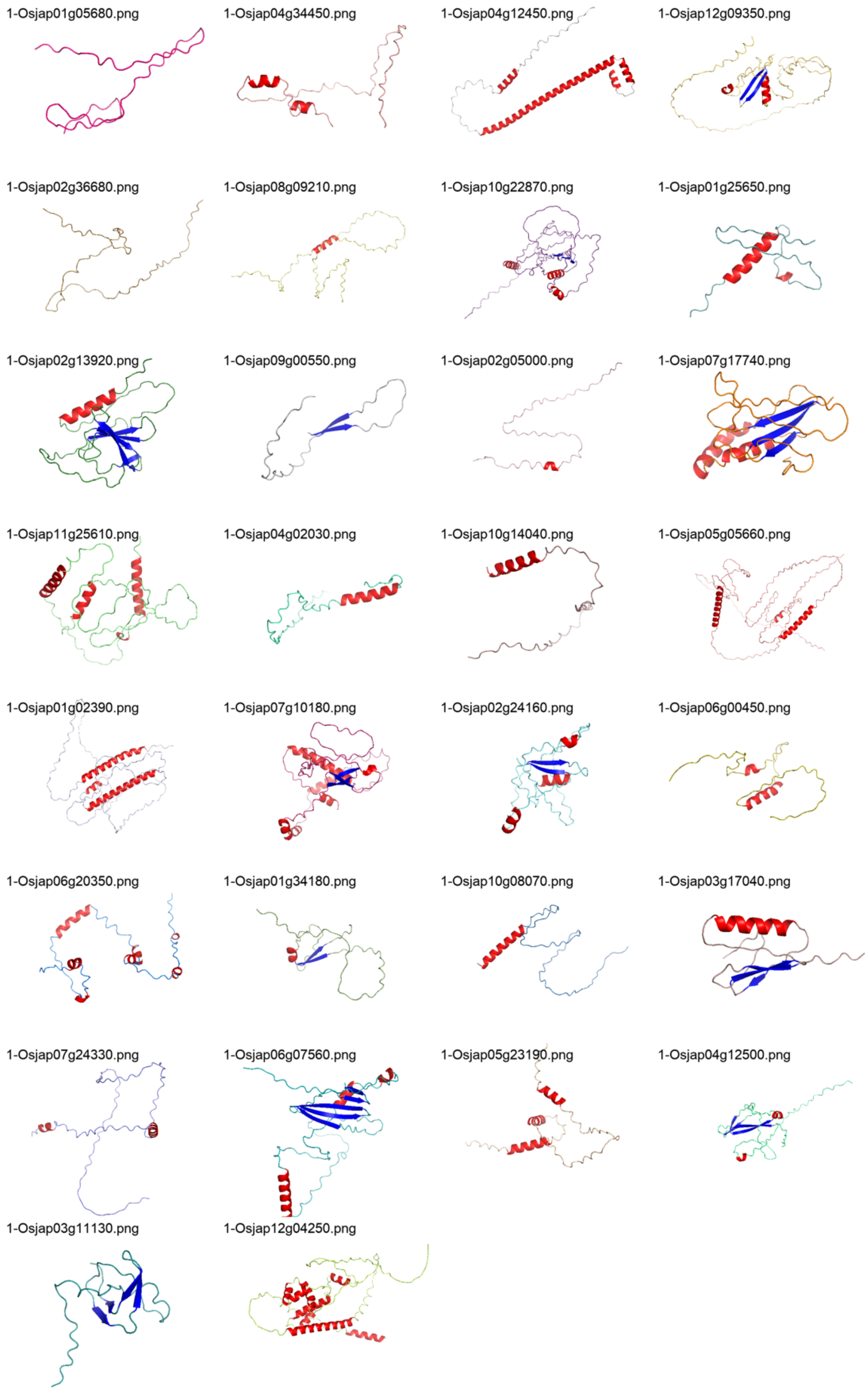

Supplementary figure 2. The protein tertiary structure of de novo genes at br1 predicted with AlphaFold2 (ranked_0). The different colors show the predicted different elements (random coil, α-helices, and β-strands). The folding qualities and categories were listed in Supplementary table 4.

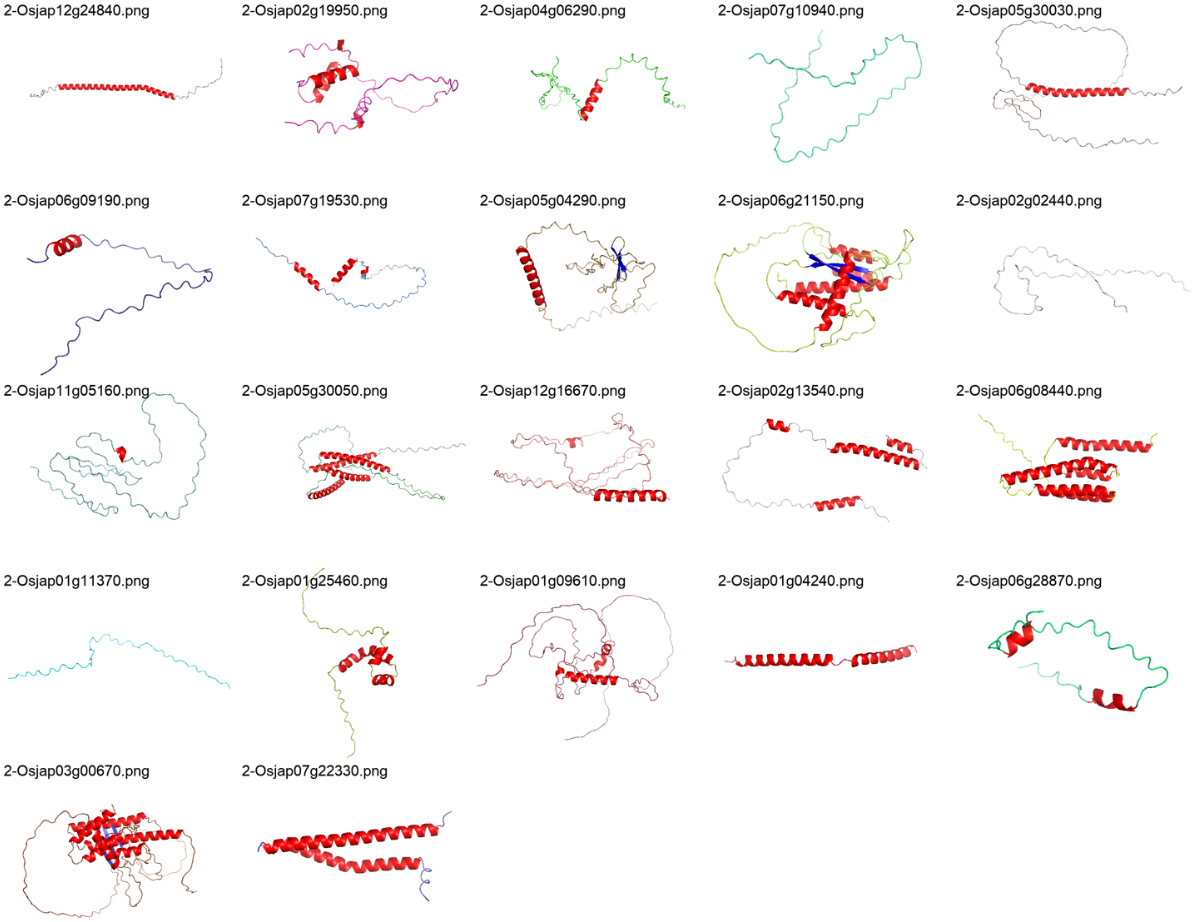

Supplementary figure 3. The protein tertiary structure of de novo genes at br2 predicted with AlphaFold2 (ranked_0). The different colors show the predicted different elements (random coil, α-helices, and β-strands). The folding qualities and categories were listed in Supplementary table 4.

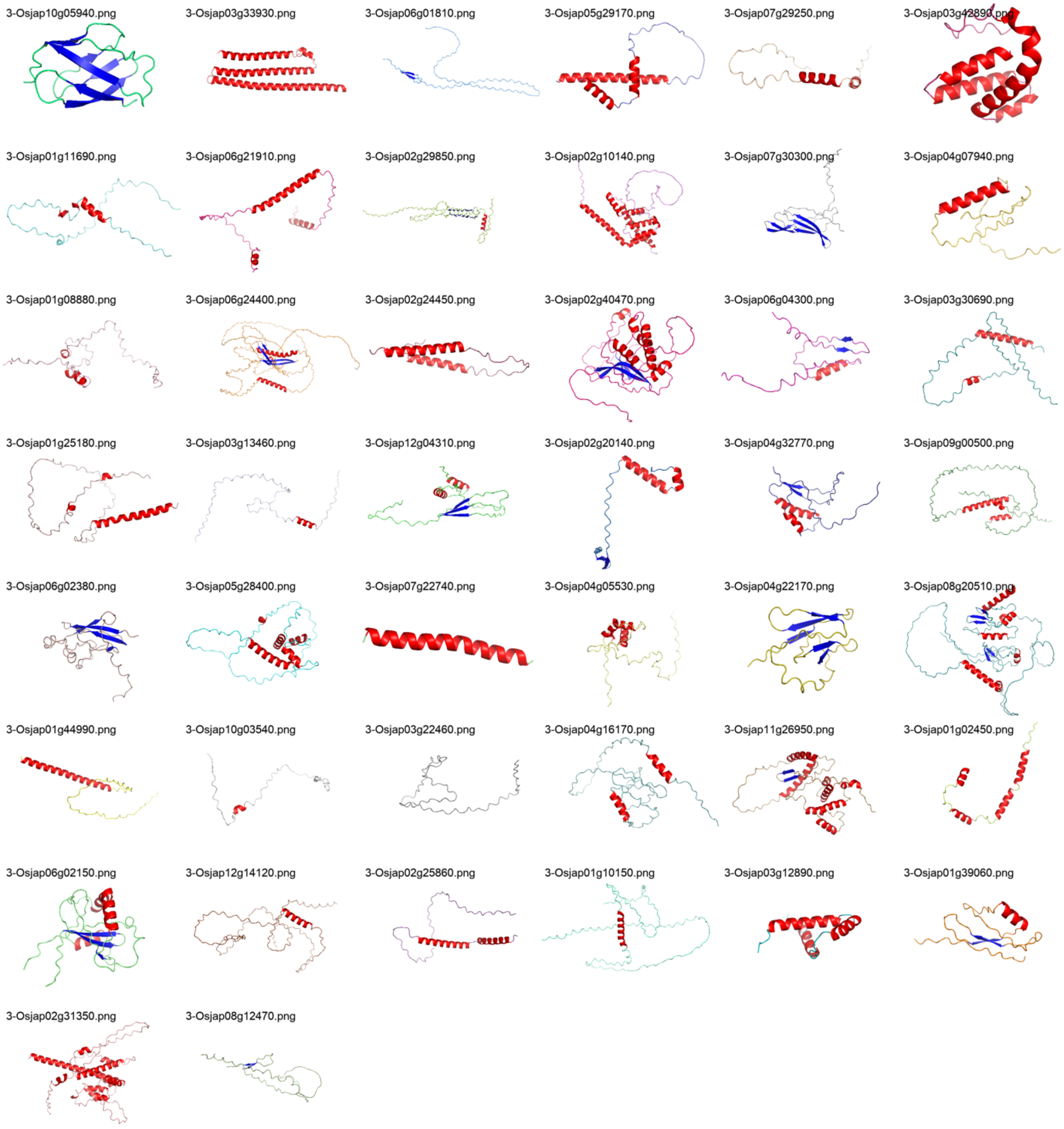

Supplementary figure 4. The protein tertiary structure of de novo genes at br3 predicted with AlphaFold2 (ranked_0). The different colors show the predicted different elements (random coil, α-helices, and β-strands). The folding qualities and categories were listed in Supplementary table 4.

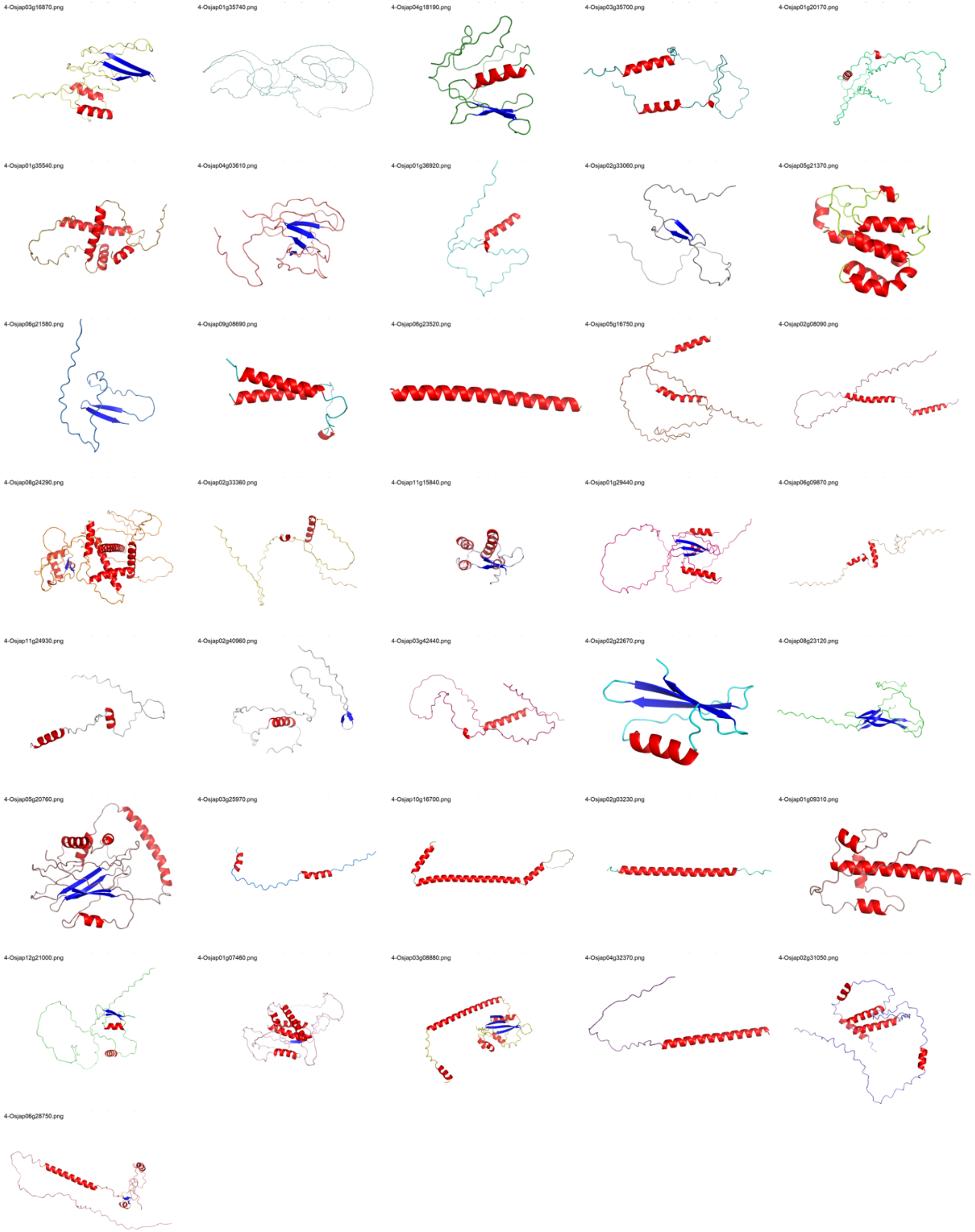

Supplementary figure 5. The protein tertiary structure of de novo genes at br4 predicted with AlphaFold2 (ranked_0). The different colors show the predicted different elements (random coil, α-helices, and β-strands). The folding qualities and categories were listed in Supplementary table 4.

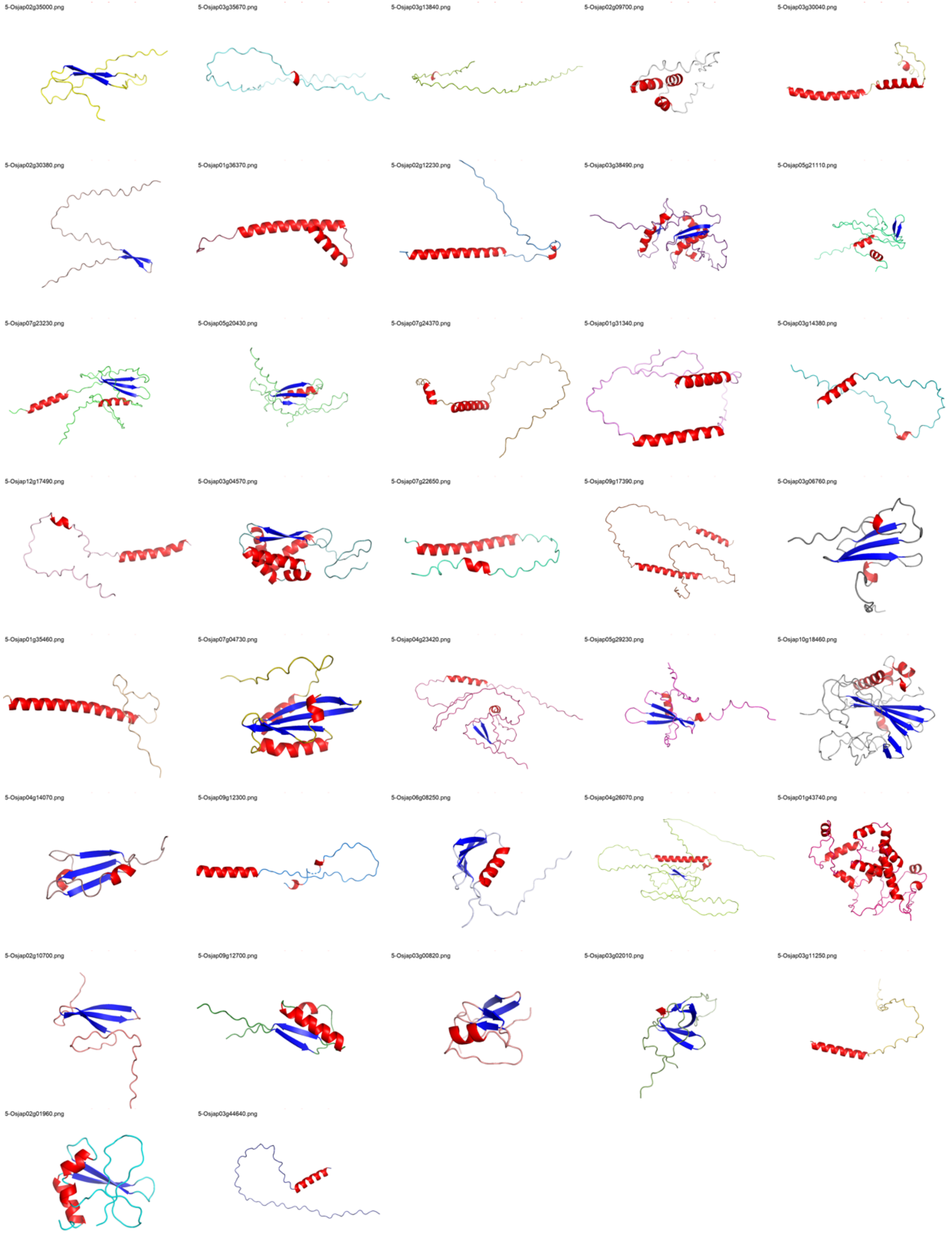

Supplementary figure 6. The protein tertiary structure of de novo genes at br5 predicted with AlphaFold2 (ranked_0). The different colors show the predicted different elements (random coil, α-helices, and β-strands). The folding qualities and categories were listed in Supplementary table 4.

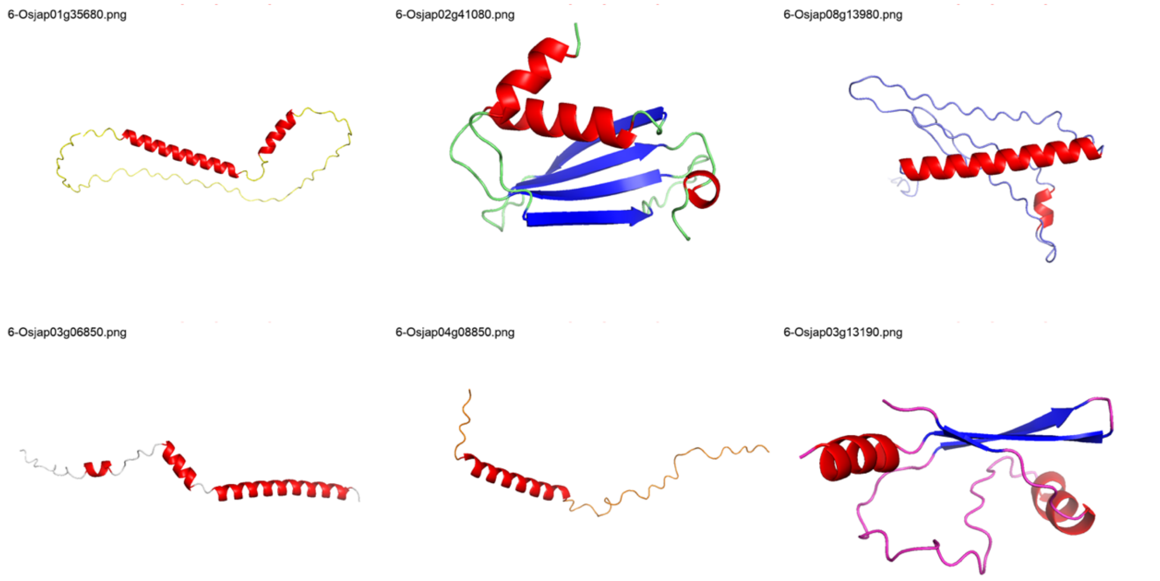

Supplementary figure 7. The protein tertiary structure of de novo genes at br6 predicted with AlphaFold2 (ranked_0). The different colors show the predicted different elements (random coil, α-helices, and β-strands). The folding qualities and categories were listed in Supplementary table 4.

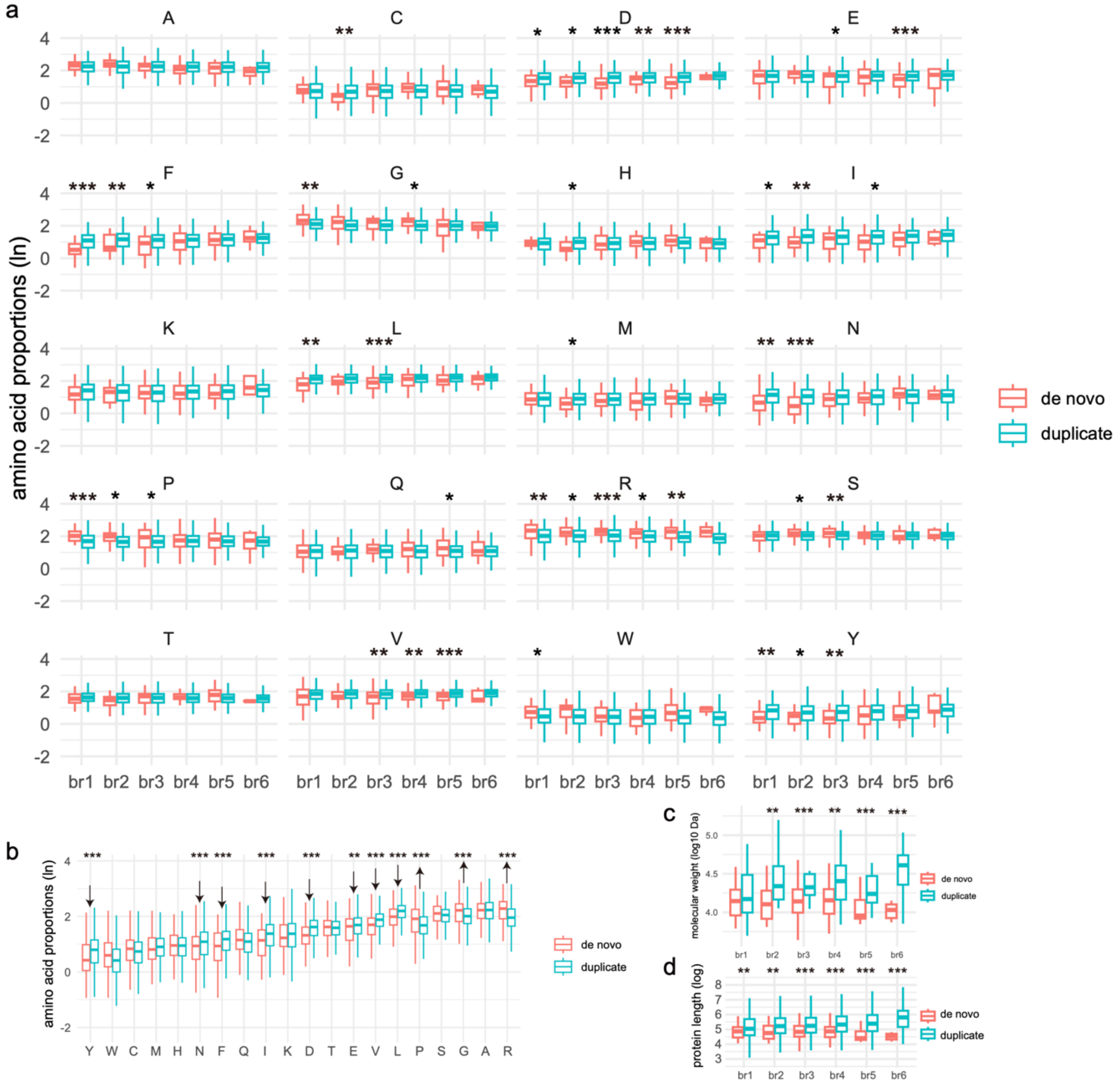

Supplementary Figure 8. Comparisons of amino acid compositions between de novo genes and gene duplicates across evolutionary branches. (a) Comparisons of the compositions (expressed as the natural logarithm of percentages) between de novo genes and gene duplicates for different amino acids within various branches. (b) Overall comparisons of compositions (natural logarithm of percentages) between de novo genes and gene duplicates for different amino acids, without age differentiation. Arrows pointing upward indicate significantly higher medians in de novo genes, and vice versa. (c) The comparisons of molecular weight (logarithm) between de novo genes and gene duplicates for genes of different ages. Note: All comparisons are based on the Wilcoxon test and only the significant pairs are shown ("*", $p < 0.05$; "**", $p < 0.01$; "***", $p < 0.001$).

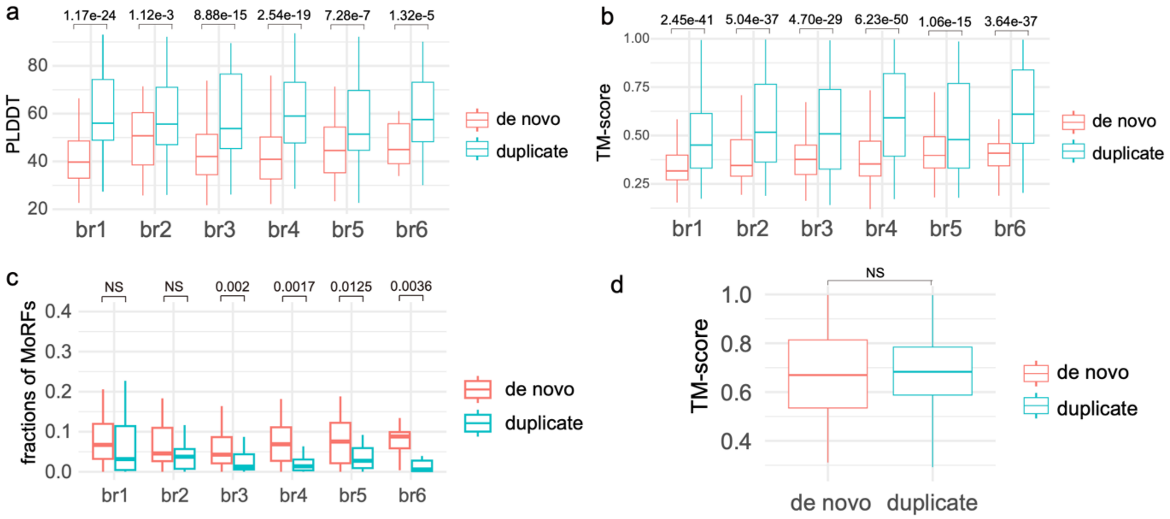

Supplementary Figure 9. Comparisons of pLDDT scores and Gibbs free energies between de novo genes and gene duplicates. (a) pLDDT score comparisons between de novo proteins and duplicates for folding predictions of isolated proteins with AlphaFold2 across evolutionary branches (ranked_0 to ranked_4). (b) Pairwise TM-scores among resulting models from Alphafold2 (ranked_0 to ranked_4) between de novo proteins and duplicates across evolutionary branches. (c) The comparisons of proportions of MoRFs between de novo proteins and duplicates with the Wilcox test (*p* values are shown above). (d) boxplot of pairwise MM-scores among resulting models from Alphafold2-multimer (all 25 models) between de novo proteins and duplicates.

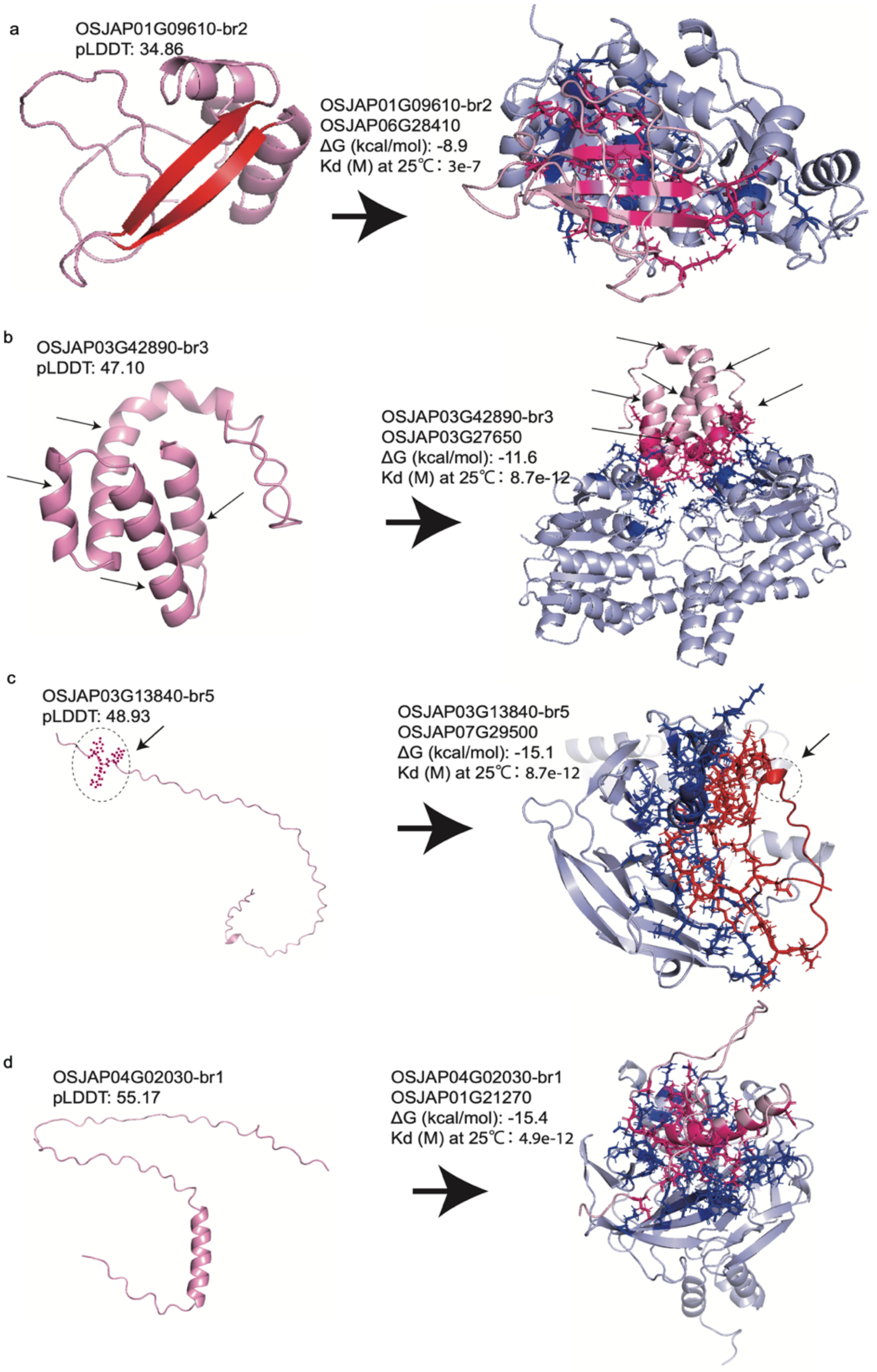

Supplementary Figure 10. The four examples of 3D structures of de novo genes and their protein complex with binding affinities (Supplementary table 6). pLDDT indicates modeling confidence for the model of ranked_0 from AlphaFold2. (a) A new β-strand appears upon binding relative to protein in isolation. (b) Two more α-helices appear in protein complex (shown with arrows). (c) The random coil segment changes into an α-helix in protein complex (shown with arrows). (d) No visible change from single protein to the complex for de novo protein.

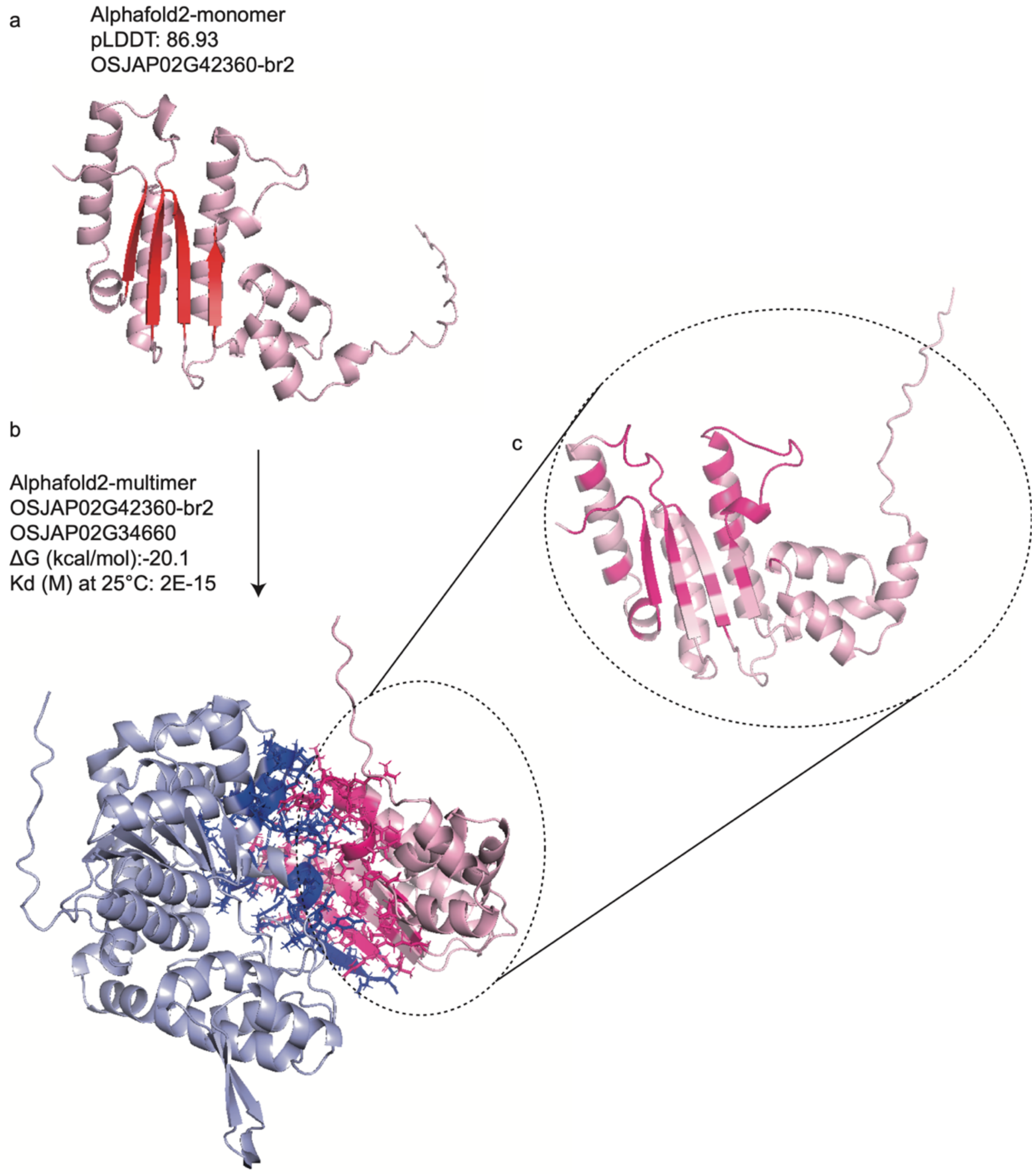

Supplementary Figure 11. The four examples of 3D structures of a duplicated protein and its protein complex with binding affinities (Supplementary table 6). (a) The monomer prediction of a duplicated protein with pLDDT showing confidence for the model of ranked_0 from AlphaFold2. (b) The predicted complex structure for the duplicated protein (pink) and its predicted partner (blue). (c) The illustration highlights the structure of the duplicated protein within the complex, with the partner component concealed. There is minimal alteration in the secondary structure when comparing the isolated protein to its form within the complex.
